# Regional and social disparities in cessation behavior and motivation to quit among U.S. adult current smokers, Tobacco Use Supplement to the U.S. Census Bureau's Current Population Survey 2014–15 and 2018–19

**DOI:** 10.3389/fpubh.2024.1416096

**Published:** 2024-10-30

**Authors:** Candon Johnson, Jose Martinez

**Affiliations:** ^1^Office Economics and Analysis, Food and Drug Administration, Silver Spring, MD, United States; ^2^Center for Tobacco Products, Food and Drug Administration, Silver Spring, MD, United States

**Keywords:** cigarettes, smoking, smoking cessation, health disparities, regional, adult, demographic, Tobacco Use Supplement

## Abstract

**Introduction:**

Variation in smoking cessation behaviors and motivators across the United States may contribute to health disparities. This study investigates regional differences over time in two key cessation motivators (quit interest and doctor's advice to quit) and two cessation behaviors (past-year quit attempts and recent successful cessation) across diverse demographic factors.

**Methods:**

Data were analyzed from two releases of the Tobacco Use Supplement to the U.S. Census Bureau's Current Population Survey (TUS-CPS) for the years 2014–15 and 2018–19. The analysis included sex, age, race and ethnicity, education, marital status, employment status, and household income.

**Results:**

Findings from 2018 to 2019 TUS-CPS revealed that quit interest was highest in the Northeast and lowest in the Midwest, while doctor's advice to quit was most prevalent in the Northeast and least in the West. Past-year quit attempts were most common in the Northeast and least in the South. Recent successful cessation (defined as quitting for 6 to 12 months) was highest in the Northeast and Midwest, with the South showing the lowest rates. Compared to the 2014–15 survey, 14 demographic groups (7 in the Midwest, 6 in the South, and 1 in the West) showed decreases in both quit interest and actions to quit. Notably, the Asian non-Hispanic group in the Northeast experienced a significant decrease in quit interest (–17.9%) but an increase in recent successful cessation (+369.2%).

**Discussion:**

Overall, the study indicates that while quit interest was highest in the West, the South exhibited the lowest rates of quit attempts and successful cessation. Significant differences were also noted between age groups. These findings highlight the need for further research into cessation behaviors at more granular levels to inform policies aimed at reducing smoking-related health disparities among populations facing the greatest challenges in cessation.

## 1 Introduction

Smoking is the leading preventable cause of death and disease in the United States, with over 480,000 premature deaths due to smoking-related illness estimated to occur each year (1). Cigarette use, including exposure to cigarette smoke, has been shown to increase the risk of cancer and imposes a substantial burden on the United States, both as an economic cost and with respect to public health (1, 2). Hence, encouraging cessation among smokers is a key method for reducing the public health burden produced by smoking. For example, quitting cigarette use can directly improve smokers' overall health and reduce their risk of premature death. In addition, cessation can add as much as a decade to life-expectancy and yields health benefits at any age (1, 3). However, despite significant decreases in the overall prevalence of cigarette smoking over time and the well-established benefits of cessation, an estimated 28.3 million (11.5%) U.S. adults ages 18 years or older reported smoking cigarettes during 2021, and cigarette smoking prevalence continues to differ by demographic and location (3, 4). While overall declines in cigarette smoking are encouraging, decreases in smoking prevalence have not been experienced equally across the United States, with some populations experiencing slower declines or increases in smoking prevalence (5, 6). Since elevated smoking prevalence among certain populations contributes to higher overall smoking prevalence, population-based methods aimed at eliminating smoking disparities also reduce the size of the overall smoking population (6). Thus, policies that seek to reduce disparities in cigarette smoking, such as targeted smoking cessation programs, also serve to hasten declines in cigarette smoking and the health burdens associated with cigarette use (7).

As national estimates indicate, most smokers have a stated desire to quit and attempt to quit cigarette use. In fact, a vast majority of smokers express some form of regret for having become a smoker, with over 71% of smokers reporting that they regretted having started smoking (8, 9). Many smokers also report a desire to quit smoking due to various reasons, such as health concerns or the financial cost of smoking (8–11). Nonetheless, only a small percentage of smokers report successfully quitting cigarette use (3, 12). This lack of efficacy among smokers is likely due to the difficulties created by cigarette dependence or other barriers to successful cessation, including nicotine withdrawal or cravings, perceived stress relief, or risk of relapse (10, 11). Higher exposure to smoking behavior within an individual's social network, such as inside a household or among friends, presents an additional barrier to those seeking to successfully quit cigarette use and increases the likelihood of relapse (13). Additional studies have indicated that over 80% of smokers report high or very high discontent due to being unable to quit, perceived addiction, and regret having started smoking (14).

In addition to these individual barriers, socioeconomic and sociodemographic disparities in quitting have been shown to contribute to existing differences in prevalence of smoking experienced among various subgroups, particularly among those with lower household incomes, those with lower education, racial and ethnic minorities, older adults, and by geography (3, 7, 15–17). Access to cessation services (e.g., counseling or cessation pharmacotherapies) and receipt of cessation advice from a medical doctor, both of which are factors associated with higher quit rates, also differ by subgroup and geographical location (3, 18–21). Furthermore, as smoking cessation is a health improving behavior, health disparities may also exist by geographical locations due to socioeconomic and sociodemographic differences in rates of quit success. Thus, cessation-related estimates at subnational levels are helpful for identifying potential health disparities across the United States and developing targeted population interventions that may provide additional increases in cessation.

This study examines changes in cessation-related prevalence (i.e., prevalence of quit interest, receipt of a doctor's advice to quit smoking, quit attempts, and successful cessation) over time among U.S. adults (18 years or older) by regional demographics. Specifically, we use the 2014–15 data release of the Tobacco Use Supplement to the Current population Survey (TUS-CPS), a supplemental survey that is administered as part of the Census Bureau's Current Population Survey, and the 2018–19 data release of the TUS-CPS to examine how cessation motivators (quit interest and receipt of a doctor's advice to quit smoking) and cessation behaviors (past-year quit attempts and recent successful smoking cessation) by sex, age, race and ethnicity, education, marital status, employment status, and annual household have progressed over time. Notably, literature on smoking cessation typically focuses on findings at a national level and the availability of subnational estimates continue to be limited (12, 15, 16, 22–24).

For example, Leventhal et al. (22), Babb et al. (12), and Trinidad et al. (24) demonstrate significant differences in cessation-related prevalence by sociodemographic at the national level, with underserved populations facing some of the highest barriers to success (12, 22, 24). Similarly, studies like those by Wang et al. (15) and Walton et al. (16) demonstrate cessation-related prevalence varies considerably according to geographic location (e.g., state and territory), while other studies such as Méndez et al. (23) have demonstrated improvements in smoking cessation over time (16, 23). While studies such as these provide helpful evidence of differences in cessation-related prevalence and progression over time, findings are rarely disaggregated further according to demographics, with estimates either being presented solely as a national level average or distinctly from geographic location. Thus, this study assists with closing a current research gap by providing information on the extent cessation-related health disparities exist among demographics at more granular levels, which is typically obscured by national and regional level averages. Additionally, given the length of time that has elapsed since the 2014–15 TUS-CPS data release (updated every 4 years) and the importance of tracking smoking cessation as a health improving behavior, this study highlights the need for further research on cessation-related prevalence among populations within each region and how disparities in smokers' cessation-related behavior have progressed over time.

## 2 Materials and methods

### 2.1 Data sources

Data was obtained from two releases of the TUS-CPS, 2014–15 and 2018–19, collected by the U.S. Census Bureau and co-sponsored by the National Cancer Institute and the Food and Drug Administration (25). The TUS-CPS provides a cross-sectional, household-based survey of noninstitutionalized U.S. civilian adults ages 18 or older across 50 states and the District of Columbia (DC). Additionally, the TUS-CPS offers a large amount of publicly available data for studying smoking cessation behaviors among U.S. adults, as well as smoking behaviors overall, at a national and regional levels (26). The TUS-CPS is conducted every 3–4 years as part of the larger Current Population Survey (CPS). Interviews were conducted by phone or in-person, with about two-thirds of respondents completing the survey by phone. During the Tobacco Use Supplement (TUS) portion of the CPS, respondents are randomly selected from each household to participate within 1 year and over three non-consecutive survey months, or waves (26). In this study, we employ the following waves of data: July 2014, January 2015, and May 2015 for the 2014–15 TUS-CPS; and July 2018, January 2019, May 2019 for the 2018–19 TUS-CPS. The number of adult self-respondents interviewed was 163,920 (average self-response rate of 54.2%) over all waves of the 2014–15 TUS-CPS and 137,471 (average self-response rate of 57.6%) over all waves of the 2018–19 TUS-CPS (25). While self-response rates varied slightly for most groups, self-response rates between those aged 18–24 and older populations represented one of the widest differences (2014–15: 33.4–65.2%; 2018–19: 35.2–64.0%). However, as noted in documentation available for the 2014–15 and 2018–19 TUS-CPS, weighting adjustments help minimize the impact of these differences on estimation. For additional information regarding self-response rates, please see the “Non-Response Bias Report” accompanying each survey available at: https://cancercontrol.cancer.gov/brp/tcrb/tus-cps. Since this study examines cessation behaviors and motivations among current and former smokers, a total of 1,512 respondents (847 during 2014–15 and 665 during 2018–19) were excluded from analysis due to indeterminate smoking status.

### 2.2 Cessation variables and statistical analysis

We follow an approach outlined by a 2019 Centers for Disease Control and Prevention (50) research brief for defining cigarette smoking cessation behaviors and cessation motivators among U.S. adults (15). Current cigarette smokers are identified as adults (ages 18 and older) who have smoked at least 100 cigarettes in their lifetimes and currently smoke every day or some days. Meanwhile, former cigarette smokers are identified as adults (ages 18 and older) who have smoked 100 cigarettes in their lifetime, but do not currently smoke at all. The first cessation motivator considered was stated interest in quitting smoking (unweighted *n* = 22,163 for the 2014–15 TUS-CPS and *n* = 15,740 for the 2018–19 TUS-CPS); it was evaluated on a scale ranging from 1 to 10 among current smokers, with 1 being not interested at all and 10 being extremely interested. Any response between 2 and 10 was used to indicate an interest in quitting. The second smoking cessation motivator considered was whether respondents received advice to quit smoking from a medical doctor. The group utilized to assess receiving advice to quit smoking from a medical doctor includes current smokers who visited a medical doctor in the past year and former smokers who indicated they visited a medical doctor within the year before they quit smoking (total unweighted *n* = 17,247 for the 2014–15 TUS-CPS and *n* = 12,224 for the 2018–19 TUS-CPS).

In addition to these cessation motivators, we examined smoking cessation behaviors, such as quit attempts and the amount of time that elapsed since an individual stopped smoking. Past-year quit attempts were defined as former smokers who quit within the past year and current smokers who either reported they stopped smoking for at least 1 day or reported that they made a serious attempt to stop smoking in the past year, regardless of the amount of time they stopped smoking (total unweighted *n* = 25,850 for the 2014–15 TUS-CPS and *n* = 18,290 for the 2018–19 TUS-CPS). The study also examines recent successful smoking cessation, which is defined as former smokers who quit within the past year and remained quit for at least 6 months. The group assessed for successful smoking cessation includes current smokers who initiated smoking 2 or more years ago and former smokers who quit smoking within the past year (total unweighted *n* = 25,507 for the 2014–15 TUS-CPS and *n* = 18,123 for the 2018–19 TUS-CPS) (27). Missing or indeterminate responses with respect to smoking status were removed from the analytical sample: 1,899 for quit interest; 1,336 for advice to quit smoking from a medical doctor; 1,067 for past-year quit attempts cessation; and 1,325 for recent successful smoking cessation.

We analyzed the effectiveness of these cessation motivators, particularly the importance of doctor advice and the smoker's desire to quit, on cessation behavior. Randomized controlled trials show that receiving doctor advice leads to a significant increase in the rate of quitting, and the effect increases as the doctor advice becomes more intensive (28). Thus, we present results beginning with cessation motivators followed by cessation behaviors.

Results are illustrated using a map of the United States and region-specific prevalence of smoking cessation motivators and behaviors using the 2014–15 and 2018–19 TUS-CPS, and the differences in prevalence between the 2014–15 and 2018–19 TUS-CPS. Demographic subgroups are categorized by the direction of change in prevalence for cessation-related behaviors and region. Demographic subgroups observing statistically significant differences in prevalence (*p* < 0.05) for each cessation-related behavior and region are separately displayed. Proxy responses (*n* = 106,435) were excluded from the analysis to reduce potential misclassifications (e.g., incorrectly answering on the behalf of another individual's smoking status)^1^. In order to account for complex survey design and yield representative estimates, data were weighted using survey provided self-response weights provided by the 2014–15 and 2018–19 TUS-CPS^2^. Rao-Scott chi-square tests were performed to determine whether differences between survey releases were statistically significant, with relative percentage changes between survey year estimates calculated manually. Statistical analysis was performed using Stata/SE, version 17.0. The appendix displays numerical results for each category at a regional and national level (Tables A4–A7). Furthermore, since differences in current cigarette use likely contribute to the variations found within this study, appendix results also include current smoking prevalence at the regional and national level (Table A3).

### 2.3 Sample demographics

Of the 299,879 adult self-respondents that indicated their smoking status during the 2014–15 and 2018–19 TUS-CPS: women represented 54.9% of the total sample; approximately 35.6% of the sample fell between the age of 45–64; and 24.9% reported a household income between $25,000 and 49,999 per year. The sample varied by race and ethnicity (Asian, non-Hispanic = 4.2%; Black, non-Hispanic = 9.8%; Hispanic = 10.6%; White, non-Hispanic = 73.0%; Other, non-Hispanic = 2.4%) and by education [12th Grade or below (No Diploma) = 9.4%; Graduation from high school = 24.5%; General Educational Diploma (GED) or equivalent = 2.9%; Some college or above = 63.2%]. Additional sample demographics, including observation counts, are presented in Table 1 and are reported by region in Table A2.

**Table 1:** Demographic characteristics of study sample, 2014–15 and 2018–19 TUS-CPS.

**Variable**	**2014–15**		**2018–19**		**Pooled**	
	**Number of observations (unweighted** ***n*****)**	**Percentage of sample (unweighted %)**	**Number of observations (unweighted** ***n*****)**	**Percentage of sample (unweighted %)**	**Number of observations (unweighted** ***n*****)**	**Percentage of sample (unweighted %)**
Total^*^	163,073	100.00%	136,806	100.00%	299,879	100.0%
**Sex**						
Male	72,946	44.7%	62,162	45.4%	135,108	45.1%
Female	90,127	55.3%	74,644	54.6%	164,771	54.9%
**Age category, years**						
18–24	10,748	6.6%	7,616	5.6%	18,364	6.1%
25–34	26,484	16.2%	21,547	15.8%	48,031	16.0%
35–44	26,909	16.5%	22,340	16.3%	49,249	16.4%
45–64	60,056	36.8%	46,757	34.2%	106,813	35.6%
65+	38,876	23.8%	38,546	28.2%	77,422	25.8%
**Race/ethnicity**						
White, non-hispanic	119,193	73.1%	99,777	72.9%	218,970	73.0%
Black, non-hispanic	16,292	10.0%	13,024	9.5%	29,316	9.8%
Hispanic	17,043	10.5%	14,729	10.8%	31,772	10.6%
Asian, non-hispanic	6,523	4.0%	5,953	4.4%	12,476	4.2%
Other, non-hispanic	4,022	2.5%	3,323	2.4%	7,345	2.4%
**Education**						
12th grade or below (no diploma)	16,536	10.1%	11,540	8.4%	28,076	9.4%
Graduation from high school	40,984	25.1%	32,598	23.8%	73,582	24.5%
GED or other equivalent	5,003	3.1%	3,810	2.8%	8,813	2.9%
Some college or above	100,550	61.7%	88,858	65.0%	189,408	63.2%
**Annual household income, $USD**						
<$25,000	38,221	23.4%	26,593	19.4%	64,814	21.6%
$25,000–49,999	41,916	25.7%	32,821	24.0%	74,737	24.9%
$50,000–74,999	30,537	18.7%	25,714	18.8%	56,251	18.8%
$75,000–99,999	19,090	11.7%	17,194	12.6%	36,284	12.1%
$100,000–149,999	18,830	11.5%	18,382	13.4%	37,212	12.4%
≥$150,000	14,479	8.9%	16,102	11.8%	30,581	10.2%
**Marital status** ^**^						
Not married	75,638	46.4%	64,763	47.3%	140,401	46.8%
Married	87,435	53.6%	72,043	52.7%	159,478	53.2%
**Employment status** ^***^						
Not working	66,275	40.6%	56,134	41.0%	122,409	40.8%
Working	96,798	59.4%	80,672	59.0%	177,470	59.2%

^*^A total of 1,512 respondents (847 during the 2014–15 TUS-CPS and 665 during the 2018–19 TUS-CPS) were excluded from analysis due to indeterminate smoking status.

^**^Marital status was defined by the variable “pemaritl” and individuals were classified as “Married” if they indicated they were married at the time of the survey, with all other respondents (e.g., divorced or widowed) being classified as “Unmarried.”

^***^Similarly, employment status was defined by the variable “prempnot,” with individuals classified as “Working” if they reported that they were employed at the time of the survey; all other respondents (e.g., unemployed or not in the labor force) were classified as “Not Working.”

## 3 Results

### 3.1 Cessation behavior and motivator prevalence from the 2014–15 TUS-CPS

We present results beginning with national estimates followed by region, and lastly demographics within regions. National estimates provide a baseline for the prevalence of smoking cessation behaviors and cessation motivators in the United States prior to presenting more granular results by region and demographics within regions. At the national level from the 2014–15 TUS-CPS, 77.5% (95% CI: 76.9–78.2%) of current smokers were interested in quitting, 71.4% (95% CI: 70.5–72.2%) received quit advice from a medical doctor in the past year, 53.5% (95% CI: 52.7–54.2%) of smokers attempted to quit, and 7.6% (95% CI: 7.2–8%) of smokers successfully quit recently.

**Figure 1:**
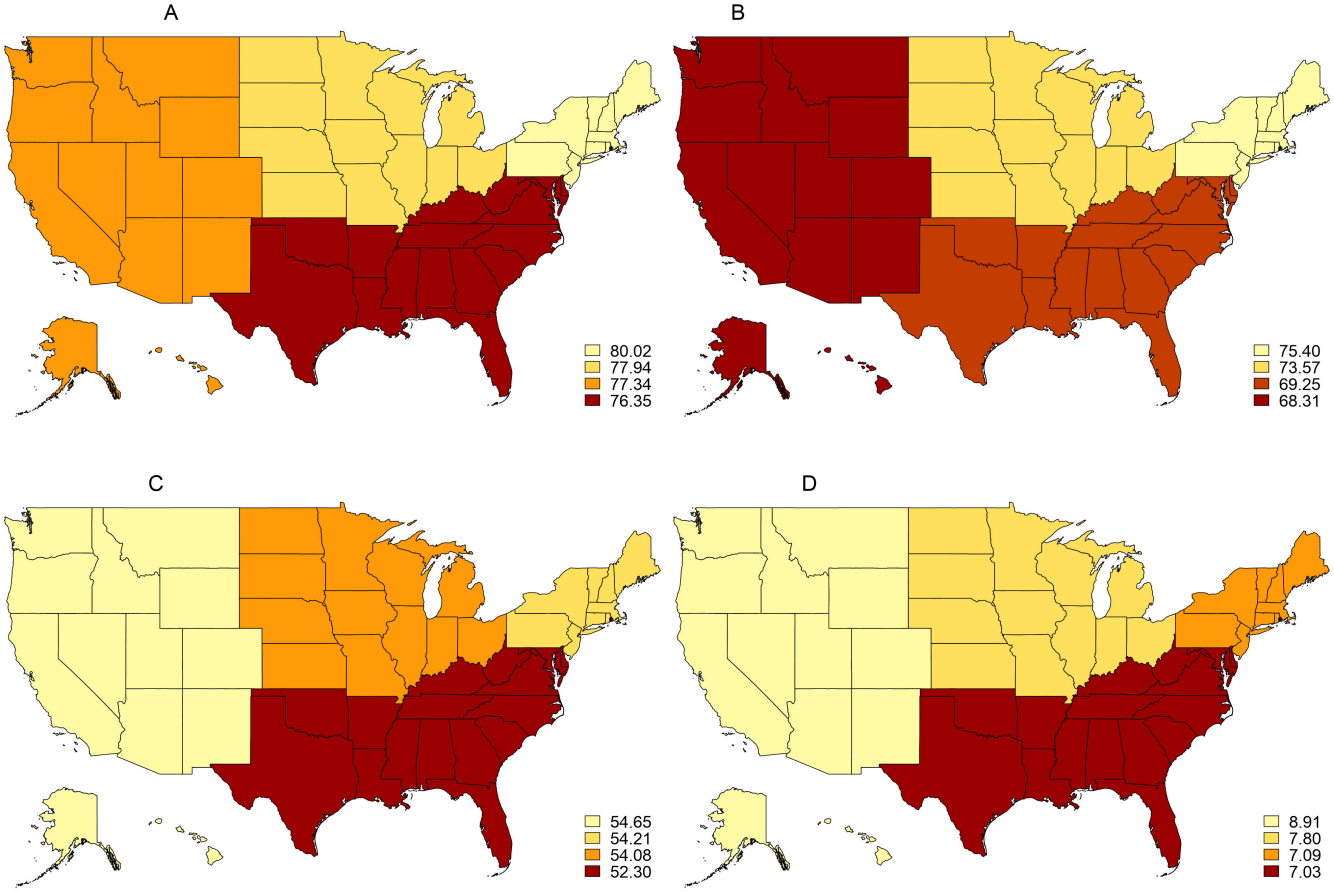
Prevalence of smoking cessation behaviors and cessation motivators among U.S. adults (18+), 2014–15 TUS-CPS. **(A)** Interested in quitting 2014/2015. **(B)** Receipt of medical doctor's advice to quilt 2014/2015. **(C)** Past-year quit attempts 2014/2015. **(D)** Recent smoking cessation 2014/2015.

This study displays the prevalence of smoking cessation behaviors and cessation motivators from the 2014–15 TUS-CPS by region (Figure 1). Over 2014–15, the Northeast region of the United States exhibited the highest rates of interest in quitting at 80.0% (95% CI: 78.3–81.6%) and receipt of quit advice from a medical doctor in the past year at 75.4% (95% CI: 73.3–77.4%). The West had the highest rates of quit attempts in the past year at 54.7% (95% CI: 52.9–56.4%) and recent successful smoking cessation at 8.9% (95% CI: 7.9–10.0%). In contrast, the South witnessed the lowest rates of interest in quitting at 76.3% (95% CI: 75.3–77.4%), making a quit attempt in the past year at 52.3% (95% CI: 51.1–53.5%), and recent successful smoking cessation at 7.0% (95% CI: 6.4–7.7%). The lowest rate of receiving advice to quit from a medical doctor was seen in the West at 68.3% (95% CI: 66.2–70.2%). For comparison, national level estimates over the same period indicate that 77.5% (95% CI: 76.9–78.2%) of respondents expressed an interest to quit, 71.4% (95% CI: 70.5–72.2%) received advice to quit from a medical doctor, 53.5% (95% CI: 52.7–54.2%) had attempted to quit, and 7.6% (95% CI: 7.2–8.0%) reported recent successful smoking cessation. Of note, although nearly one in eight smokers reported an interest in quitting during the 2014–15 TUS-CPS, less than one-tenth reported abstaining from cigarette use for a period longer than 6 months. The discrepancies between smokers' desire to quit, quit attempts, and recent successful quitting are representative of the addictive nature of smoking and the associated difficulties of quitting cigarette use. States included within each region are displayed within Table A1.

Table 2 reports the highest and lowest prevalence of smoking cessation behaviors and Table 3 reports the highest and lowest prevalence for cessation motivators within each demographic by region from the 2014–15 TUS-CPS. Among all demographics, prevalence of quit interest was found to be generally highest in the Northeast and lowest in the West and South, with reported interest to quit falling between 62.0 and 91.6%. With respect to race and ethnicity, people from the Asian, non-Hispanic group in the Northeast had the highest interest in quitting, whereas people who identify as Other, non-Hispanic in the West expressed the lowest quit interest. By household income, Northeastern households within the topmost income bracket ($150,000 or above) reported the highest interest to quit. In contrast, Western households within the lowest income (below $25,000) expressed the least interest in quitting. Similarly, those within the youngest age demographic (18–24 years old) in the Northeast were the most interested in quitting, with the oldest age demographic (65 years or older) in the West having the least interest in quitting. Individuals with the highest educational attainment (at least some college education or more) in the Northeast were the most interested in quitting and those with the lowest educational attainment [12th grade or below (no diploma)] in the South were the least interested. Lastly, quit interest was found to be highest among the working, married, and female populations in the Northeast and lowest among working, unmarried, and male populations located in the South.

**Table 2:** Highest and lowest cessation motivator prevalence within each demographic, TUS-CPS 2014–15.

**Quit interest**			**Doctor's advice to quit**		
**Demographic subgroup**	**Region**	**% (95% CI)**	**Demographic subgroup**	**Region**	**% (95% CI)**
**Highest**					
Asian, non-hispanic	Northeast	91.6 (80.9, 96.6)	12th grade or below (no diploma)	Northeast	81.7 (76.2, 86.1)
$150,000+	Northeast	88.6 (81.6, 93.1)	65+	Northeast	81.6 (76.9, 85.5)
18–24 years old	Northeast	84.6 (76.9, 90.1)	Black, non-hispanic	Midwest	78.4 (73.1, 82.9)
Some college or above	Northeast	82.9 (80.4, 85.2)	Not working	Midwest	78.0 (75.7, 80.2)
Working	Northeast	81.6 (79.3, 83.6)	$50,000–74,999	Northeast	77.6 (72.9, 81.7)
Female	Northeast	81.2 (78.8, 83.3)	Female	Northeast	76.1 (73.3, 78.7)
Married	Northeast	80.6 (77.9, 83.1)	Married	Northeast	75.8 (72.6, 78.7)
**Lowest**					
65+	West	62.0 (56.8, 66.8)	18–24 years old	West	46.4 (37.9, 55.1)
Other/non-hispanic	West	70.0 (62.8, 76.3)	Hispanic	South	57.2 (50.9, 63.2)
12th grade or below (no diploma)	South	70.7 (68.1, 73.2)	$100,000–149,999	West	63.6 (55.5, 71.1)
Not working	South	73.1 (71.5, 74.7)	Working	West	65.7 (62.8, 68.5)
Below $25,000	West	74.4 (71.6, 77.1)	12th grade or below (no diploma)	West	66.1 (59.8, 71.8)
Male	South	75.3 (73.8, 76.8)	Not married	West	66.5 (63.9, 69.1)
Not married	South	76.3 (74.9, 77.6)	Female	West	67.9 (65.1, 70.6)

For reference, the national averages for Quit Interest and Doctor's Advice to Quit are 77.5% (95% CI: 76.9–78.2%) and 71.4% (95% CI: 70.5–72.2%), respectively; regional averages for Quit Interest are 80.0% (95% CI: 78.3–81.6%) in the Northeast, 77.9% (95% CI: 76.6–79.2%) in the Midwest, 76.3% (95% CI: 75.3–77.4%) in the South, and 77.3% (95% CI: 75.8–78.9%) in the West; and regional averages for Doctor's Advice to Quit are 75.4% (95% CI: 73.3–77.4%) in the Northeast, 73.6% (95% CI: 72.0%, 75.1%) in the Midwest, 69.2% (95% CI: 67.9%, 70.6%) in the South, and 68.3% (95% CI: 66.2%, 70.3%) in the West.

**Table 3:** Highest and lowest cessation behavior prevalence within each demographic, TUS-CPS 2014–15.

**Quit attempts**			**Recent successful quitting**		
**Demographic subgroup**	**Region**	**% (95% CI)**	**Demographic subgroup**	**Region**	**% (95% CI)**
**Highest**					
18–24 years old	West	65.5 (59.8, 70.9)	$150,000+	Midwest	16.3 (10.5, 24.4)
Other/non-hispanic	Midwest	63.8 (55.4, 71.4)	18–24 years old	West	13.1 (9.4, 18)
$150,000+	Northeast	62.5 (54.4, 69.9)	Hispanic	Midwest	12.9 (8.4, 19.4)
Not working	Northeast	58.8 (55.8, 61.7)	Some college or above	West	10.5 (9.1, 12.2)
GED or other equivalent	Midwest	57.9 (52.1, 63.4)	Married	West	9.7 (8.1, 11.6)
Female	Northeast	57.6 (55, 60.3)	Working	West	9.6 (8.3, 11.1)
Married	West	55.0 (52.2, 57.7)	Female	West	9.3 (7.9, 11)
**Lowest**					
65+	West	45.1 (40.5, 49.7)	GED or other equivalent	South	3.1 (2, 4.8)
Asian, non-hispanic	Northeast	46.4 (34.8, 58.5)	Asian, non-hispanic	Northeast	3.7 (1.1, 11.4) ^*^RSE
12th grade or below (no diploma)	South	47.5 (44.8, 50.2)	Below $25,000	Northeast	3.9 (2.9, 5.3)
$150,000+	South	50.1 (43.1, 57.2)	45–64 years old	South	5.6 (4.8, 6.5)
Male	South	50.4 (48.7, 52)	Not working	South	5.8 (5, 6.7)
Not working	South	50.9 (49.1, 52.6)	Not married	South	6.3 (5.6, 7.2)
Not married	South	51.4 (49.9, 52.9)	Male	South	6.6 (5.8, 7.5)

Due to low precision, estimates with a relative standard error (RSE) > 30% have been indicated by “^*^RSE” and should be interpreted with caution. For reference, the national averages for Quit Attempts and Recent Successful Quitting are 53.5% (95% CI: 52.7–54.2%) and 7.6% (95% CI: 7.2–8.0%), respectively; regional averages for Quit Attempts are 54.2% (95% CI: 52.3–56.1%) in the Northeast, 54.1% (95% CI: 52.7–55.5%) in the Midwest, 52.3% (95% CI: 51.1–53.5%) in the South, and 54.7% (95% CI: 52.9–56.4%) in the West; and regional averages for Recent Successful Quitting are 7.1% (95% CI: 6.2–8.1%) in the Northeast, 7.8% (95% CI: 7.0%, 8.6%) in the Midwest, 7.0% (95% CI: 6.4%, 7.7%) in the South, and 8.9% (95% CI: 7.9%, 10.0%) in the West.

Prevalence of advice to quit from a medical doctor was generally highest in the Northeast among individuals with the lowest educational attainment, those in the oldest age group, females, married respondents, and households with an income of $50,000–74,999. Quit advice prevalence was also found to be higher in the Midwest among Black, non-Hispanic respondents and those not working. The lowest quit advice prevalence was observed mostly among demographic subgroups in the West and included individuals with the lowest educational attainment, despite being among one of the subgroups with the highest quit advice prevalence in the Northeast. Other demographic subgroups with lower quit advice prevalence included those within the youngest age group, working respondents, those not married, and females in the West, with the remaining subgroup being Hispanic individuals in the South.

Similar to quit interest, quit attempts were generally highest in the Northeast among the youngest age group, those within the highest income bracket, females, and married individuals. Prevalence was also highest among GED recipients, those not working, and people from the Other, non-Hispanic group in the Northeast and Midwest. Interestingly, although the highest earning demographic in the Northeast reported the highest quit interest, Southern households in the same household income bracket exhibited the lowest rate of quit attempts. Quit attempts were generally found to be lowest in the South and among the oldest age demographic, those with the least education, males, those not working, those not married, and those who identify as Asian, non-Hispanic.

With respect to recent quit success, prevalence was highest in the West and Midwest among the highest income households, those with the most education, the youngest age group, those working, married individuals, females, and people from the Hispanic group. On the other hand, recent successful quitting was generally lowest in the South among those with a GED or equivalent, the oldest age group, individuals reporting that they were not working, those reporting they were unmarried, and males. Lower recent successful quitting prevalence was also observed in the Northeast among households in the lowest income bracket and people from the Asian, non-Hispanic group.

### 3.2 Cessation behavior and motivator prevalence from the 2018–19 TUS-CPS

At the national level from the 2018–2019 TUS-CPS, 76.6% (95% CI: 75.8–77.4%) of current smokers were interested in quitting, 71.8% (95% CI: 70.8–72.8%) received quit advice from a medical doctor in the past year, 51.9% (95% CI: 51–52.8%) of smokers attempted to quit, and 7.4% (95% CI: 6.9–7.9%) of smokers successfully quit recently.

**Figure 2:**
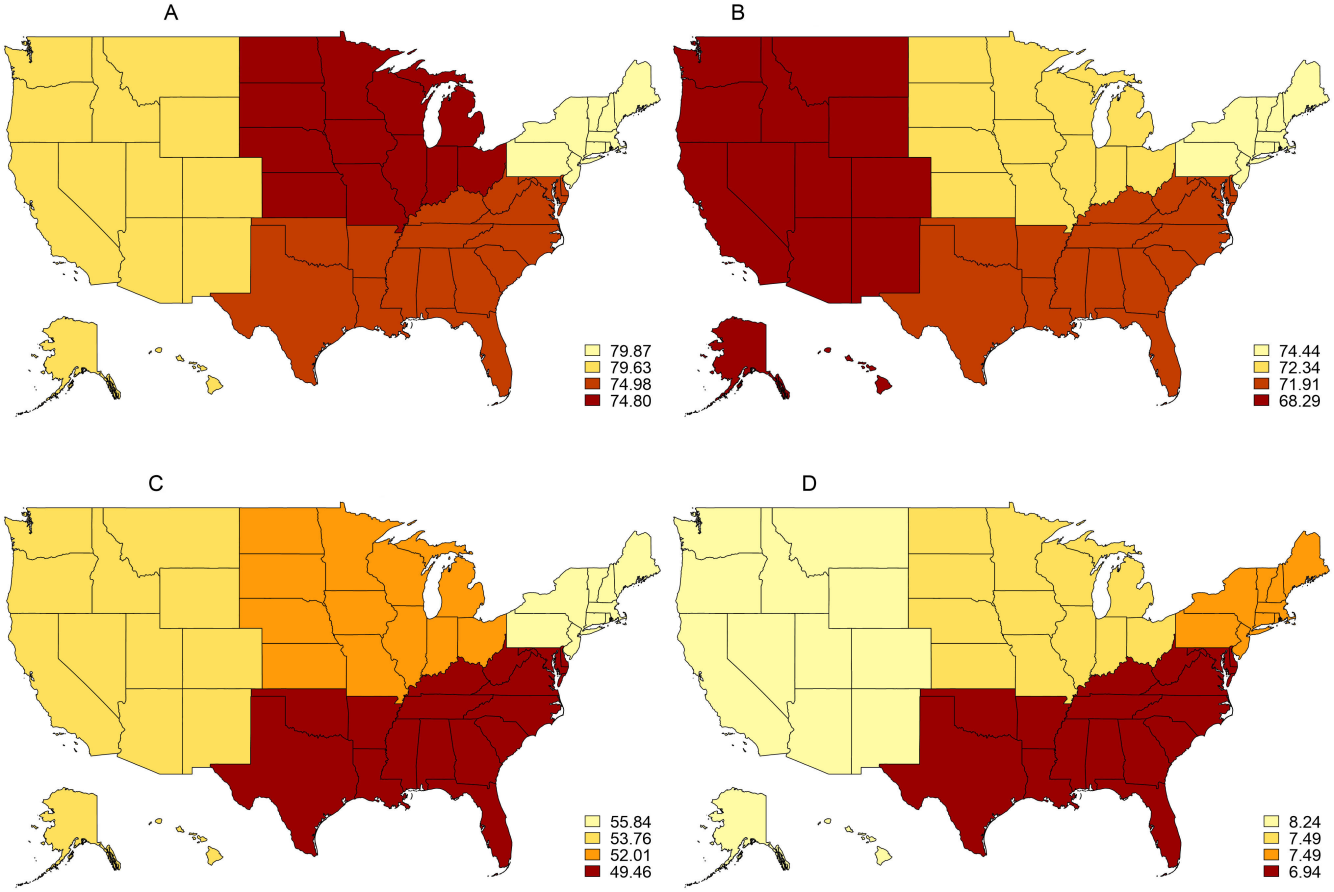
Prevalence of smoking cessation behaviors and cessation motivators among U.S. adults (18+), 2018–19 TUS-CPS. **(A)** Interested in quitting 2018/2019. **(B)** Receipt of medical doctor's advice to quilt 2018/2019. **(C)** Past-year quit attempts 2018/2019. **(D)** Recent smoking cessation 2018/2019.

Figure 2 displays the prevalence of smoking cessation behaviors and cessation motivators using data from the 2018–19 TUS-CPS. The Northeast region of the United States exhibited the highest rates of interest in quitting at 79.9% (95% CI: 77.8–81.8%), receiving quit advice from a medical doctor in the past year at 74.4% (95% CI: 71.9–76.9%), and quit attempts in the past year at 55.8% (95% CI: 53.5–58.1%). The West saw the highest rates of recently quitting smoking at 8.2% (95% CI: 7.2–9.5%). The South saw the lowest rates of making a quit attempt in the past year at 49.5% (95% CI: 48.1–50.9%), and recently quitting smoking at 6.9% (95% CI: 6.2–7.7%). The lowest rate of receiving advice to quit from a medical doctor was seen in the West at 68.3% (95% CI: 65.7–70.7%), while the Midwest had the lowest rate of interest in quitting at 74.8% (95% CI: 73.1–76.2%). At the national level, reported quit interest was 76.6% (95% CI: 75.8–77.4%), receipt of advice to quit from a medical doctor was 71.8% (95% CI: 70.8–72.8%), attempts to quit was 51.9% (95% CI: 51.0–52.8%), and recent successful quitting was 7.4% (95% CI: 6.9–7.9%). As was noted with the 2014–15 TUS-CPS, the discrepancy between an expressed interest in quitting smoking by current smokers and successful quitting by former smokers has persisted.

Table 4 reports the highest and lowest prevalence of smoking cessation behaviors and Table 5 reports the highest and lowest prevalence for cessation motivators by demographic and region from the 2018–19 TUS-CPS. Among all demographics, prevalence of quit interest was found to be generally highest in the West and lowest in the Midwest and South, with reported interest to quit falling between 65.1 and 89%. With respect to race and ethnicity, those who identify as Black, non-Hispanic in the Northeast had the highest interest in quitting, whereas those who identify as Hispanic in the Midwest expressed the lowest quit interest. By household income, Southern households within the uppermost income bracket ($150,000 or above) reported the highest interest to quit, while households expressing the least interest in quitting fell within the lowest income bracket (below $25,000) and were also located in the South. Representing a similar contrast, the youngest age demographic (18–24 years old) in the West was the most interested in quitting, while the oldest age demographic (65 years or older) in the South was the least interested in quitting. With respect to education, individuals with at least some college education in the West were the most interested in quitting, while 12th grade or below (no diploma) in the Midwest were the least interested. Lastly, quit interest was found to be highest among the working, unmarried, and female populations located in the West and lowest among non-working, unmarried, and male individuals in the South.

**Table 4:** Highest and lowest cessation motivator prevalence within each demographic, TUS-CPS 2018–19.

**Quit interest**			**Doctor's advice to quit**		
**Demographic subgroup**	**Region**	**% (95% CI)**	**Demographic subgroup**	**Region**	**% (95% CI)**
**Highest rates**					
18–24 years old	West	89.0 (82.7, 93.2)	65+	Northeast	81.9 (77, 86)
$150,000+	South	88.3 (82.6, 92.2)	$150,000+	Northeast	81.1 (71.8, 87.9)
Black, non-hispanic	Northeast	87.0 (80.1, 91.8)	Asian, non-hispanic	West	79.7 (69.6, 87.1)
Some college or above	West	82.7 (80.5, 84.8)	12th grade or below (no diploma)	Northeast	77.7 (69.8, 83.9)
Working	West	82.0 (79.8, 84.1)	Not working	Northeast	77.4 (73.6, 80.7)
Not married	West	80.8 (78.5, 82.8)	Married	Northeast	75.6 (71.3, 79.5)
Female	West	80.7 (78.2, 83)	Female	Northeast	74.5 (71.1, 77.5)
**Lowest rates**					
12th grade or below (no diploma)	Midwest	65.1 (59.6, 70.2)	18–24 years old	Northeast	52.2 (35.9, 68)
65+	South	65.5 (62.2, 68.7)	Hispanic	West	59.2 (52.1, 65.9)
Hispanic	Midwest	67.2 (56, 76.8)	$150,000+	South	62.4 (53.3, 70.7)
Not working	Midwest	71.7 (68.9, 74.4)	Not married	West	66.5 (63.1, 69.7)
Below $25,000	South	73.2 (71, 75.3)	Male	West	66.9 (63.2, 70.4)
Male	Midwest	73.5 (71, 75.8)	Working	West	66.9 (63.4, 70.3)
Not married	South	74.5 (72.9, 76.1)	12th grade or below (no diploma)	West	67.2 (58.5, 75)

For reference, the national averages for Quit Interest and Doctor's Advice to Quit are 76.6% (95% CI: 75.8–77.4%) and 71.8% (95% CI: 70.8–72.8%), respectively; regional averages for Quit Interest are 79.9% (95% CI: 77.8–81.8%) in the Northeast, 74.8% (95% CI: 73.1–76.4%) in the Midwest, 75.0% (95% CI: 73.7–76.2%) in the South, and 79.6% (95% CI: 77.8–81.3%) in the West; and regional averages for Doctor's Advice to Quit are 74.4% (95% CI: 71.9–76.9%) in the Northeast, 72.3% (95% CI: 70.3%, 74.3%) in the Midwest, 71.9% (95% CI: 70.3%, 73.5%) in the South, and 68.3% (95% CI: 65.8%, 70.7%) in the West.

**Table 5:** Highest and lowest cessation behavior prevalence within each demographic, TUS-CPS 2018–19.

**Quit Attempts**			**Recent successful quitting**		
**Demographic subgroup**	**Region**	**% (95% CI)**	**Demographic subgroup**	**Region**	**% (95% CI)**
**Highest rates**					
Black, non-hispanic	Northeast	67.3 (59.5, 74.3)	Hispanic	Midwest	17.5 (11.1, 26.5)
18–24 years old	Midwest	65.9 (57.4, 73.6)	18–24 years old	West	14.7 (9.8, 21.5)
$75,000–99,999	Northeast	60.5 (53.5, 67.2)	$150,000+	Northeast	12.3 (7.3, 20)
12th grade or below (no diploma)	Northeast	58.0 (51.4, 64.2)	Some college or above	West	9.7 (8.2, 11.5)
Female	Northeast	57.8 (54.6, 61)	Working	West	9.0 (7.5, 10.7)
Not married	Northeast	56.7 (53.8, 59.5)	Married	Midwest	8.4 (7, 10.1)
Working	Northeast	56.1 (53, 59.1)	Male	West	8.2 (6.8, 9.9)
**Lowest rates**					
65+	West	45.0 (40.4, 49.7)	GED or other equivalent	South	3.5 (2, 5.9)
12th grade or below (no diploma)	Midwest	45.4 (40, 50.8)	Black, non-hispanic	Midwest	3.7 (1.9, 7)^*^RSE
$150,000+	Midwest	45.6 (37.1, 54.3)	$25,000–49,999	Northeast	4.0 (2.6, 6.1)
Male	South	46.2 (44.2, 48.2)	45–64 years old	South	4.6 (3.8, 5.6)
Married	South	47.5 (45.3, 49.8)	Not working	South	5.5 (4.7, 6.5)
White, non-hispanic	South	47.9 (46.3, 49.5)	Male	South	6.6 (5.6, 7.6)
Not working	South	49.4 (47.3, 51.4)	Not married	South	6.7 (5.8, 7.7)

Due to low precision, estimates with a relative standard error > 30% have been indicated by “^*^RSE” and should be interpreted with caution. For reference, the national averages for Quit Attempts and Recent Successful Quitting are 51.9% (95% CI: 51–52.8%) and 7.4% (95% CI: 6.9–7.9%), respectively; regional averages for Quit Attempts are 55.8% (95% CI: 53.5–58.1%) in the Northeast, 52.0% (95% CI: 50.2–53.8%) in the Midwest, 49.5% (95% CI: 48.1–50.9%) in the South, and 53.8% (95% CI: 51.7–55.8%) in the West; and regional averages for Recent Successful Quitting are 7.5% (95% CI: 6.3–8.9%) in the Northeast, 7.5% (95% CI: 6.6%, 8.5%) in the Midwest, 6.9% (95% CI: 6.2%, 7.7%) in the South, and 8.2% (95% CI: 7.2%, 10.0%) in the West.

Prevalence of advice to quit from a medical doctor was generally highest in the Northeast among individuals with the lowest educational attainment (12th grade or below, without a high school diploma), those in the oldest age group, females, those not working, married respondents, and households reporting an income of $150,000 or more. Quit advice prevalence was also found to be higher in the West among the Asian, non-Hispanic respondents. Demographic subgroups having the lowest quit advice prevalence were primarily located in the West among individuals with the lowest educational attainment (12th grade or below, without a high school diploma), working respondents, those reporting they were unmarried, males, and those who identify as Hispanic. Lower quit advice prevalence was also observed among the youngest age demographic in the Northeast and Southern households having an income of $150,000 or more.

Quit attempt prevalence was predominantly highest in the Northeast among Black, non-Hispanic respondents, females, unmarried individuals, and those not working. Respondents also reporting relatively high prevalence of quit attempts in the Northeast were those having the lowest educational attainment and those with a household income of $75,000–99,999. Prevalence was also highest in the Midwest among the youngest respondents (18–24 years old). On the other hand, lower attempts to quit was observed largely among participants located in the South and Midwest. In the Southern region, quit attempt prevalence was found to be lower among males, those not working, unmarried individuals, and White, non-Hispanic respondents. In the Midwest, the highest earning and least educated demographics were among those with the lowest quit attempt prevalence. In the West, those falling within the oldest age demographic reported the overall lowest prevalence of quit attempts.

While recent quit success prevalence continues to be substantially lower than other cessation metrics, respondents located in the West and Midwest generally reported the highest rates of success, with those who identify as Hispanic in the Midwest having the overall highest reported quit success (nearly one in five). Among other demographics reporting relatively higher quit success prevalence were those with the highest educational attainment, falling within the youngest age demographic (18–24 years old), working, and males in the West; and married individuals and households falling within the highest income bracket ($150,000 or more) in the Midwest. In contrast, demographics located in the Southern region of the United States reported the lowest quit success when compared to other regions. In particular, recent successful quitting prevalence was lowest among recipients of a GED or equivalent degree, the 45–64-year-old age demographic, males, those not working, and unmarried individuals. Prevalence was also lowest for Black, non-Hispanic individuals in the Midwest, and individuals with a household income of $25,000–49,999 in the Northeast. Those with a GED or equivalent degree in the South had the lowest rate of quit success when compared to all other regional demographics, with <1 in every 25 respondents reporting successful cessation in the past year.

### 3.3 Changes in cessation behavior and motivator prevalence between the 2014–15 TUS-CPS and 2018–19 TUS-CPS

This study examines the percentage change in cessation-related prevalence between the 2014–15 TUS-CPS and 2018–19 TUS-CPS. At the national level, we find quit attempts decreased by 2.9% between these two surveys. National changes for interest in quitting, the receipt of advice to quit from a medical doctor, and recent successful cessation did not fall within a level of statistical significance (*p* < 0.05).

**Figure 3:**
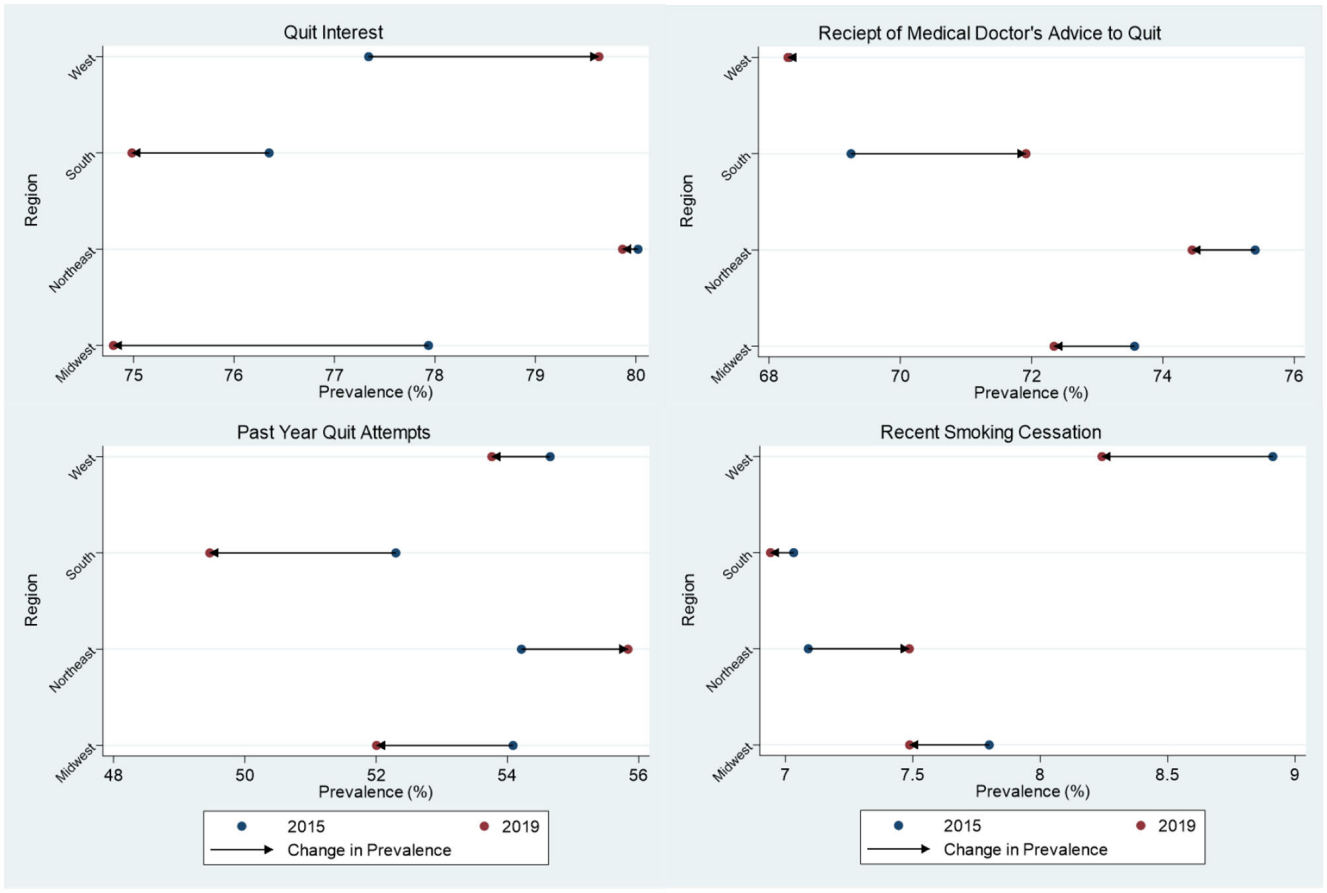
Differences in prevalence of smoking cessation behaviors and cessation motivators among US adults (18+) between the 2014–15 and 2018–19 TUS-CPS. Statistically significant changes include an increase in advice to quit from a medical doctor in the South at the 0.05 level, and a decrease in interest in quitting in the Midwest at the 0.01 level.

Figure 3 displays the regional-level percentage change in prevalence for cessation motivators and cessation behaviors since the 2014–15 survey release. Compared to 2014–15, the Northeast and West 2018–19 results did not show significant changes in cessation motivation or behavior. No regions saw statistically significant increases in interest in quitting. The South witnessed the only significant movement in receiving advice to quit from a medical doctor, with an increase of 3.85% (*p* = 0.0130). However, this region also observed the largest decrease in quit attempts at 5.42% (*p* = 0.0020). The Midwest experienced the largest decrease in interest in quitting at 4.02% (*p* = 0.0030) and a corresponding, though not statistically significant, decrease in quit attempts at 3.84% (*p* = 0.0740). No significant changes in recent successful quitting prevalence were observed at the regional level when compared to the 2014–15 survey release. At the national level, quit interest declined by 1.24% (*p* = 0.0694), doctor's advice to quit was nearly unchanged with a 0.62% increase (*p* = 0.5110), past-year quit attempts decreased by 2.9% (*p* = 0.0086), and recently quitting smoking declined by 2.06% (*p* = 0.6280). However, we note that the national difference in prevalence for past-year quit attempts was the only cessation-related metric that fell within a level of statistical significance.

**Figure 4:**
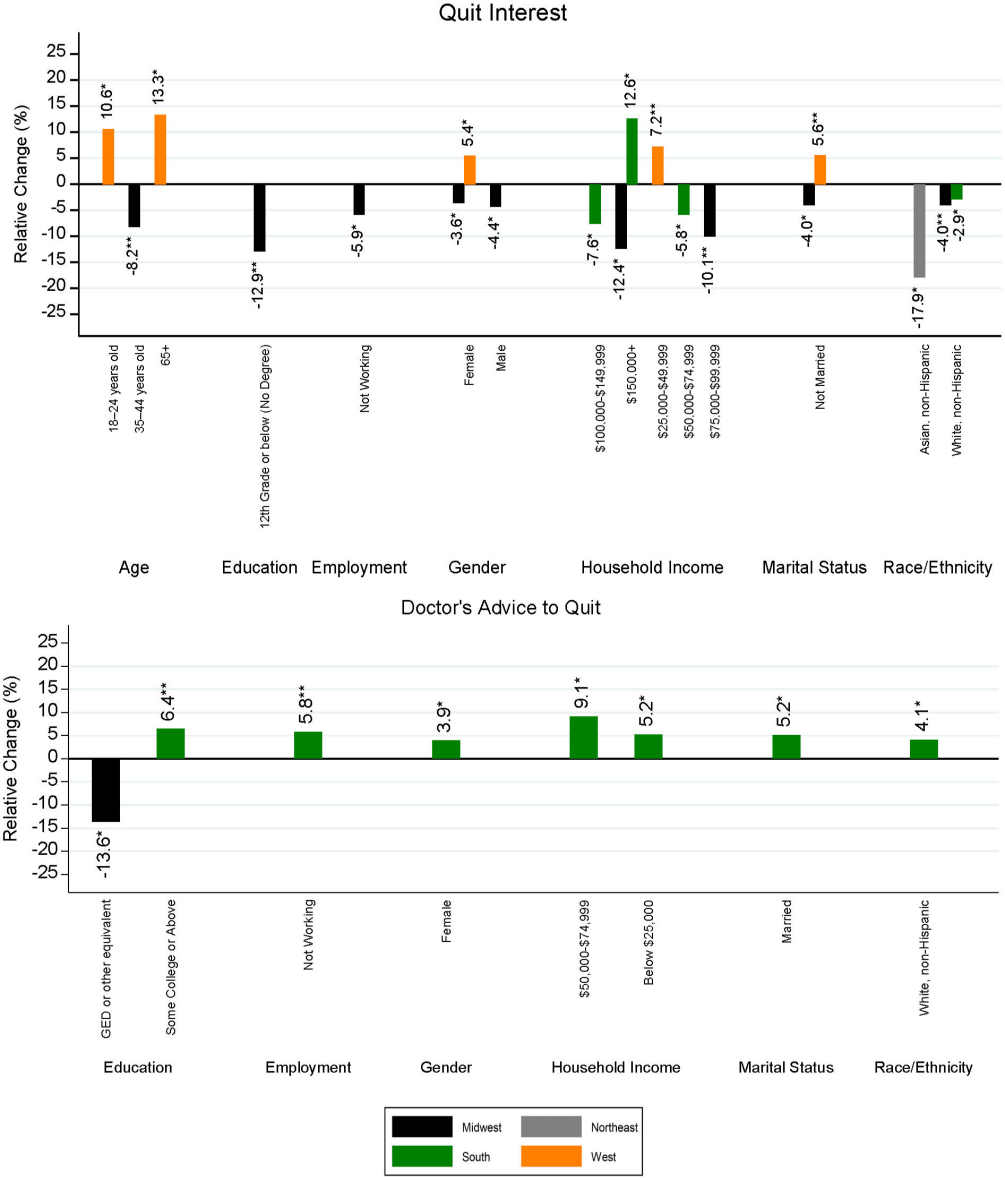
Statistically significant differences in regional smoking cessation motivators by demographic subgroup and region between the 2014–15 and 2018–19 TUS-CPS. The level of significance for differences between survey years is indicated as follows: **p < 0.01, and *p < 0.05.

**Figure 5:**
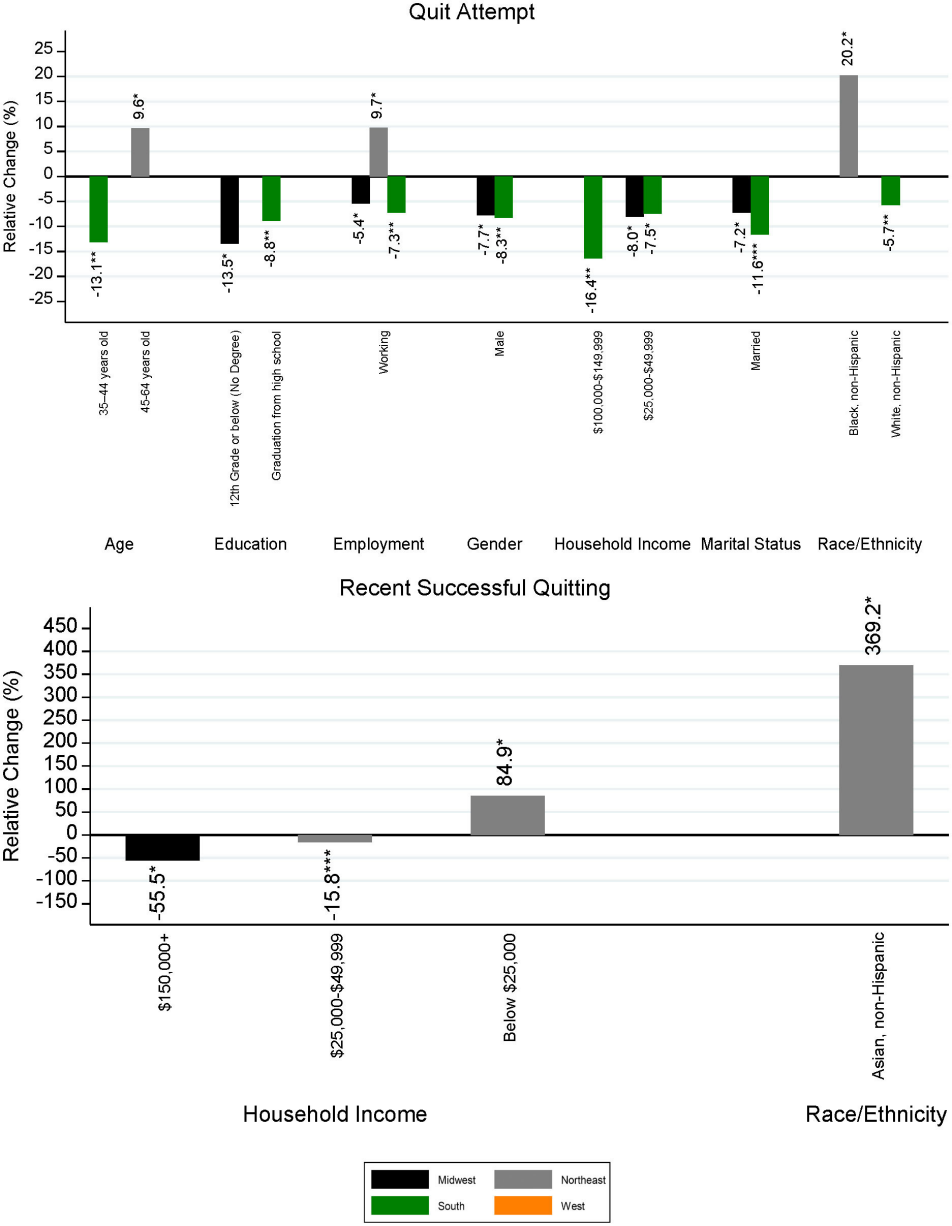
Statistically significant differences in regional smoking cessation behaviors by demographic subgroup and region between the 2014–15 and 2018–19 TUS-CPS. The level of significance for differences between survey years is indicated as follows: ***p < 0.001, **p < 0.01, and *p < 0.05.

Regional demographics that were found to have statistically significant differences in cessation-related prevalence between the 2014–15 and 2018–19 surveys, along with their estimated changes in prevalence, are presented in Figure 4 (prevalence of smoking cessation motivators) and Figure 5 (prevalence of smoking cessation behaviors). With respect to quit interest, the largest significant decrease was experienced among people from the Asian, non-Hispanic group in the Northeast (−17.9%; *p* = 0.0392) and the largest significant increase in quit interest was experienced among individuals ages 65 or older (+13.3%; *p* = 0.0178) in the West. Significant declines in quit interest were experienced mostly by demographic subgroups in the Midwest, particularly by sex, marital status, and among every educational subgroup except those with a high school diploma. The West had the largest number of significant increases in quit interest, with no observable declines among demographic subgroups in this region. Movements among some demographic subgroups also differed by region. When compared to the Midwest, females and unmarried individuals located in the West observed slightly larger improvements in quit interest. Similarly, while individuals with the highest household income ($150,000 or more) in the Midwest observed a 12.4% decline (*p* = 0.0445) in quit interest, a nearly equal increase (+12.6%; *p* = 0.0191) was experienced by this same subgroup in the South.

As for doctor's advice to quit, significant differences were mostly positive and occurred largely among individuals in the South, with no significant changes between the two survey periods being observed in the Northeast or West. The largest increase in quit advice was experienced among households having an income level between $50,000 and 74,999 in the South (+9.1%; *p* = 0.0109). On the other hand, households in the South falling under the lowest income bracket (below $25,000) witnessed a moderate boost (+5.2%; *p* = 0.0318) in quit interest. The largest decrease occurred among those with a GED or equivalent in the Midwest (−13.6%; *p* = 0.0262), with an increase among those having an educational background of some college or above in the South (+6.4%; *p* = 0.0073).

With respect to quit attempts, the Northeast was the only region to experience increases in prevalence between the 2014–15 and 2018–19 surveys, and there were no observable decreases in quit attempt prevalence. Significant changes in the South and Midwest were found to be negative, representing fewer quit attempts in these regions. No significant changes were observed in the West. The largest increase in quit attempts was among people from the Black, non-Hispanic group in the Northeast (+20.2%; *p* = 0.0228), whereas the largest decrease was found among Southern households having an income of $100,000–149,999 (−16.4%, *p* = 0.0062). Among married individuals, declines were seen within every region except the Northeast, with the largest decrease observed in the South (−11.6%, *p* < 0.0001). Two demographic subgroups were found to have opposite movements by region. Individuals ages 35–44 in the South experienced a 13.1% decline in reported quit attempts (*p* = 0.0011), whereas those located in the Northeast saw a 9.6% increase (*p* = 0.0309). Among the working population, those in the Northeast witnessed a 9.7% (*p* = 0.0138) increase in quit attempts, while individuals located in the Midwest and South underwent a decline of 5.4% (*p* = 0.0450) and 7.3% (*p* = 0.0020), respectively.

Recent successful quitting had the least number of significant changes when comparing the 2014–15 and 2018–19 surveys, with changes occurring mostly in the Northeast and South according to household income. However, the magnitude of changes falling within statistical significance was larger relative to the other examined cessation-related variables of this study. The largest increase in recent successful cessation was observed among the Asian, non-Hispanic population in the Northeast, having an increase of 369.2% (*p* = 0.0484) or nearly 4 times that of 2014–15, and the largest decrease (−55.5%, *p* = 0.0115) was found among Midwestern households with an income of $150,000 or greater (the highest income bracket). On the other hand, those in the lowest income group, reporting a household income of $25,000 or less, experienced an increase of 84.9% (*p* = 0.0212) in successful quitting. While statistically significant, changes in prevalence for the Asian, non-Hispanic population and households with an income of $150,000 or greater incorporated estimates with low precision (i.e., a high RSE) and had relatively small sample sizes in both the 2014–15 and 2018–19 TUS-CPS. The magnitude of changes for these demographic groups should be interpreted with caution; however, we present estimates with relatively low sample size to provide additional information and highlight the need to examine under sampled subgroups of interest for future research.

Finally, to separate a stated desire to quit smoking from actions taken to quit smoking, demographic subgroups within each region were categorized by the direction of change in prevalence for quit interest and the direction of change for quit attempts and recent successful cessation when comparing the 2014–15 and 2018–19 survey results. Black, non-Hispanic individuals in the Northeast were the only demographic to experience an increase in quitting interest and an increase in taking actions to quit, while White, non-Hispanic individuals in the West were the only demographic to experience an increase in quitting interest but a decrease in taking actions to quit. A total of 14 demographic subgroups (seven in the Midwest; six in the South; and one in the West) observed decreases in both quitting interest and taking actions to quit. Demographics experiencing declines in both quit interest and actions taken to quit varied by age, education, employment, and income across the Midwest and South. The most common demographic subgroups witnessing declines in both quit interest and actions taken to quit were males in the Midwest and South and married individuals in the Midwest, South, and West. No demographic subgroups experienced a decrease in quitting interest and an increase in actions to quit.

## 4 Discussion

Data analyzed from TUS-CPS show slight decreases in overall quit interest, quit attempts, and recent smoking cessation between 2014–15 and 2018–19 and a small increase in the receipt of a medical doctor's advice to quit smoking. Cessation-related prevalence declined in the time period between the two survey collections for most demographic subgroups, with the largest number of declines observed in the Midwest and the largest number of increases occurring in the South. The Midwest was the only region to experience no statistically significant improvements in cessation-related behavior. Additionally, demographic subgroups in the Northeast had the largest number of increases and the lowest number of declines with respect to cessation-related prevalence.

Using the 2018–19 TUS-CPS, we find that the prevalence of cessation motivators and behaviors vary considerably by demographic subgroup at the regional level, a result that is consistent with other studies observing similar variation among demographics at the national level and by geographic location (12, 15, 16, 22–24). Additionally, as compared to the 2014–15 TUS-CPS, a wide range of outcomes were observed when examining differences in regional-level prevalence of cessation motivators and behaviors by demographic subgroup. While statistically significant improvements in cessation-related prevalence were observed among demographic subgroups in the Northeast, South and West, the Midwest witnessed no significant improvements. In fact, when examined at the demographic level, the Midwest region had the largest number of demographic subgroups observing a decline in cessation prevalence, especially with respect to quit interest. While not specifically assessed, regional-level differences found in this study may be due to factors such as changes in tobacco control programs, cigarette taxes, availability of cessation services or treatments, access to healthcare, indicators of addiction, or type of cigarette used (menthol or non-menthol) (3, 22, 29–38). For example, while interest to quit increased among ages 18–24 in the West when compared to the 2014–15 survey release, this same age group experienced a significant increase in recent successful cessation in the South but no statistically significant difference in the prevalence for past-year quit attempts in all regions.

Although a measure of addiction was not directly explored by this study, the large discrepancy between the desire to quit expressed by survey participants and actions taken to quit across all regions and demographics is especially suggestive of the addictive nature of cigarette use. National estimates during 2014–15 show nearly eight in 10 smokers have an expressed interest to quit smoking, whereas fewer than one in 10 former smokers reported successfully quitting for at least 6 months (15). Our findings demonstrate these large discrepancies between an expressed desire to quit and actualized cessation behavior persist at granular levels, vary by region and demographic, and likely contribute to the high levels of regret experienced by smokers who are unable to achieve successful cessation (8, 9, 14). For example, over 2014–15 the Asian, non-Hispanic population in the Northeast had the highest quit interest prevalence among all demographic subgroups; however, this same population also had one of the lowest prevalence for quit attempts and recent successful quitting. Additionally, while reported quit interest over 2018–19 was generally highest among demographic subgroups in the Western region, those with the lowest prevalence of quit attempts and successful quitting were typically located within the South.

These results also demonstrate how the intensity of addiction may increase as smoking persists over the lifetime, with older populations finding quitting especially challenging. As shown in Tables 2, 3, stark differences in quit interest and recent successful cessation were found to exist between the oldest and youngest age groups of our sample. Quit interest prevalence among those aged 18–24 in the Northeast was found to be 22.6 percentage points higher than among those aged 65 or older in the West. Similarly, quit attempt prevalence among those aged 18–24 in the West was 20.5 percentage points higher than among those aged 65 or older in the West. With respect to recent successful cessation prevalence, the prevalence among ages 18–24 in the West was found to be nearly double than of those aged 45–64 in the South. While individuals aged 18–24 generally have higher successful cessation, quit attempts, and a desire to quit, this age group had the lowest receipt of advice to quit from a medical doctor in the West, representing an area for improvement. Thus, while national estimates are informative, findings such as these highlight the importance of examining cessation behaviors and motivators at levels typically obscured by national averages.

Furthermore, observed differences in cessation behaviors and motivation may contribute to health disparities experienced by various demographic subgroups within each region, particularly among populations having the lowest rates of cessation or motivators that encourage cessation (1). While cessation at younger ages is associated with lower risk of premature death due to smoking-related disease (e.g., quitting before age 40 reduces the risk of premature death associated with continued smoking by around 90%), we find that doctor's advice to quit smoking was lowest among those of age 18–24 when compared to other age groups, regardless of region (39, 40). In particular, the 2018–19 survey found that the disparity in prevalence of advice to quit was widest between individuals aged 65 or older (81.9%) and aged 18–24 (52.2%) in the Northeast, representing a nearly 30 percentage point difference between these groups. Since cessation at younger ages would provide the largest mortality benefits, this discrepancy in quit advice highlights an area for potential improvement. Additionally, although the highest prevalence of quit attempts was among the non-Hispanic Black population in the Northeast, the non-Hispanic Black population was found to have some of the lowest rates of recent successful cessation, particularly within the Midwest and South. Further demonstrating the influence of geography, while higher prevalence for quit advice was observed among those within lowest educational attainment in the Northeast during the 2018–19 TUS-CPS, individuals having similar educational attainment in the West reported one of the lowest prevalence for quit advice. This contrast was also observed among households falling within the highest income bracket, with those in the Northeast reporting higher quit advice and those in the South having lower reported quit advice. By examining changes in smoking cessation behaviors since the 2014–15 release of the TUS-CPS, these findings provide examples of where additional targeted efforts may further reduce health disparities among certain populations and thereby gain reductions in the overall health burden associated with smoking.

### 4.1 Limitations

Our findings are subject to several limitations. First, data are self-reported and thus results may be subject to response bias (e.g., social desirability bias or recall bias) and/or other measurement error. Additionally, self-response rates varied by demographic group, with those aged 18–24 and older populations having the widest difference. However, we note that the CPS employs data collection methods and complex survey design in order to mitigate potential measurement errors and ensure data quality (51, 52). Survey weights are also supplied to help minimize the impact of self-response rates on estimation. Second, due to the cross-sectional design of the survey, causation or temporality cannot be established between cessation motivators and cessation behaviors. Third, while remaining quit over 6–12 months significantly reduces the odds of relapse, the definition of recent successful cessation may include some individuals who may return to smoking over a longer period of time (41, 42). Additionally, multiple quit attempts made by individuals are not considered within this analysis and may potentially translate into higher levels of sustained cessation over time (43). Fourth, sample size was limited for several demographic subgroups and may impact the precision of some estimates by region. Thus, while the interpretation of these estimates should be taken with caution, we choose to present estimates with relatively low sample size to provide additional information and highlight the need to examine under sampled subgroups of interest for future research. Fifth, this study only examines cessation with respect to cigarettes rather than complete tobacco cessation. For example, we did not account for co-use of other tobacco products or the possibility of switching from cigarettes to other tobacco products, such as electronic cigarettes, vaping products, or other electronic nicotine delivery system products, when ceasing cigarette use. Therefore, our findings should be considered within the context of cigarette cessation. Changes in tobacco policies (e.g., the introduction of smoke-free laws, increased spending on cessation interventions, or changes in tobacco taxation) were not assessed between the 2014–15 and 2018–19 TUS-CPS data releases and may have contributed to observed differences in prevalence for smoking cessation and motivation to quit at regional levels (33–36, 38, 44). Lastly, data were examined through May 2019 and findings do not include impacts from the COVID-19 pandemic.

## 5 Conclusion

National level findings do not reveal the wide range of cessation behaviors among regional demographics. Wide discrepancies between reported interest in quitting, past-year quit attempts, and past-year successful smoking cessation were found to exist among each demographic subgroup at the national and regional levels, and likely influence unequal declines in cigarette smoking prevalence experienced across the United States. Compared to the 2014–15 survey information, the 2018–19 data shows that demographic subgroups in the Midwest and South witnessed the largest number of declines in quitting behavior, particularly in quit interest and quit attempts. While differences between current smokers' desire to quit and recent successful quitting could be due to several reasons, such as changes in tobacco policies, levels of addiction, cessation methods used to quit, type of cigarette smoked (menthol vs. non-menthol), or number of quit attempts needed before reaching sustained cessation, exploring the contribution of these factors at more granular levels represents an area for future investigation.

Our results fill a gap in the literature by providing estimates of cessation-related behaviors among demographics within regions and how these behaviors have changed across time. In addition, these results may provide a resource to inform targeted efforts, such as improving discrepancies in quit advice and coverage of cessation programs, that could help improve smoking cessation rates, and thereby mitigate health disparities among populations that face the highest barriers for progress (3, 24, 44–49). These findings could also be used to inform distributional analyses conducted as part of rulemaking. Distributional analyses determine how regulatory impacts are experienced by various population groups across the United States and whether some groups are impacted differently than others. Increasing cessation-related efforts in areas of the United States facing particularly low rates of successful cessation, may lead to further reductions in the death and disease related to tobacco use and health disparities experienced by populations facing the highest barriers to cessation.

## Data Availability

Publicly available datasets were analyzed in this study. This data can be found here: https://cancercontrol.cancer.gov/brp/tcrb/tus-cps.

## Footnotes

^1^Proxy responses were collected whenever a survey-eligible individual was unable to be reached for self-interview. Proxy respondents represent a single individual (included as a member of the household and over the age of 15) that answers on the behalf of all eligible members of a household.

^2^Self-response weights adjust for both non-response and self-response. The non-response adjustment accounts for occupied households that completed and responded to the CPS but did not complete the TUS questionnaire. The self-response adjustment accounts for the exclusion of interviews completed by proxy. Further explanation of survey weights are available in the technical documentation for the 2014-15 and 2018-19 TUS-CPS. Please see: https://cancercontrol.cancer.gov/brp/tcrb/tus-cps.
