# Supplementary material for: Regional and social disparities in cessation behavior and motivation to quit among U.S. adult current smokers, Tobacco Use Supplement to the U.S. Census Bureau's Current Population Survey 2014–15 and 2018–19
Source: Front Public Health. 2024 Oct 30;12:1416096. doi: 10.3389/fpubh.2024.1416096 (PMC11557426; doi:10.3389/fpubh.2024.1416096)
Supplement: Supplementary file 1 [file Table_1.DOCX]

Supplementary Material

Appendix to Regional and Social Disparities in Cessation Behavior and Motivation to Quit among U.S. Adult Current Smokers, Tobacco Use Supplement to the U.S. Census Bureau’s Current Population Survey 2014-15 and 2018-19

Candon Johnson^1^, Jose Martinez^2*^

^1^Office of Economics and Analysis, Food and Drug Administration, Silver Spring, MD

^2^Center for Tobacco Products, Food and Drug Administration, Silver Spring, MD

***Correspondence:** Corresponding Author: [jose.martinez@fda.hhs.gov](mailto:jose.martinez@fda.hhs.gov)

# Appendix Table 1. States by Region

| **Region** | **States** |
| --- | --- |
| Northeast | CT, MA, ME, NH, NJ, NY, PA, RI, VT |
| South | AL, AR, DC, DE, FL, GA, KY, LA, MD, MS, NC, OK, SC, TN, TX, VA, WV |
| Midwest | IA, IL, IN, KS, MI, MN, MO, ND, NE, OH, SD, WI |
| West | AK, AZ, CA, CO, HI, ID, MT, NM, NV, OR, UT, WA, WY |

# Appendix Table 2. Demographic Characteristics of Study Sample by Region, 2014-15 and 2018-19 TUS-CPS.

| **Variable** | **Northeast** | | **Midwest** | | **South** | | **West** | |
| --- | --- | --- | --- | --- | --- | --- | --- | --- |
|  | **Number of Observations (Unweighted n)** | **Percentage of Sample (Unweighted %)** | **Number of Observations (Unweighted n)** | **Percentage of Sample (Unweighted %)** | **Number of Observations (Unweighted n)** | **Percentage of Sample (Unweighted %)** | **Number of Observations (Unweighted n)** | **Percentage of Sample (Unweighted %)** |
| Total* | 50,016 | 100.00% | 63,325 | 100.00% | 109,473 | 100.00% | 77,065 | 100.00% |
| Sex | | | | | | | | |
| Male | 22,469 | 44.92% | 28,902 | 45.64% | 48,378 | 44.19% | 35,359 | 45.88% |
| Female | 27,547 | 55.08% | 34,423 | 54.36% | 61,095 | 55.81% | 41,706 | 54.12% |
| Age Category, Years | | | | | | | | |
| 18–24 | 2,377 | 4.75% | 3,880 | 6.13% | 6,983 | 6.38% | 5,124 | 6.65% |
| 25–34 | 7,058 | 14.11% | 10,245 | 16.18% | 17,858 | 16.31% | 12,870 | 16.70% |
| 35–44 | 7,722 | 15.44% | 10,073 | 15.91% | 17,874 | 16.33% | 13,580 | 17.62% |
| 45-64 | 18,945 | 37.88% | 22,555 | 35.62% | 38,590 | 35.25% | 26,723 | 34.68% |
| 65+ | 13,914 | 27.82% | 16,572 | 26.17% | 28,168 | 25.73% | 18,768 | 24.35% |
| Race/Ethnicity | | | | | | | | |
| White, non-Hispanic | 39,914 | 79.80% | 53,859 | 85.05% | 73,713 | 67.33% | 51,484 | 66.81% |
| Black, non-Hispanic | 3,366 | 6.73% | 4,054 | 6.40% | 19,544 | 17.85% | 2,352 | 3.05% |
| Hispanic | 4,050 | 8.10% | 2,864 | 4.52% | 11,317 | 10.34% | 13,541 | 17.57% |
| Asian, non-Hispanic | 2,135 | 4.27% | 1,407 | 2.22% | 2,869 | 2.62% | 6,065 | 7.87% |
| Other, non-Hispanic | 551 | 1.10% | 1,141 | 1.80% | 2,030 | 1.85% | 3,623 | 4.70% |
| Education | | | | | | | | |
| 12th Grade or below (No Diploma) | 4,103 | 8.20% | 4,656 | 7.35% | 12,522 | 11.44% | 6,795 | 8.82% |
| Graduation from high school | 12,852 | 25.70% | 16,495 | 26.05% | 27,751 | 25.35% | 16,484 | 21.39% |
| GED or equivalent | 1,222 | 2.44% | 1,734 | 2.74% | 3,755 | 3.43% | 2,102 | 2.73% |
| Some college or above | 31,839 | 63.66% | 40,440 | 63.86% | 65,445 | 59.78% | 51,684 | 67.07% |
| Annual Household Income, $USD | | | | | | | | |
| <25,000 | 9,554 | 19.10% | 12,619 | 19.93% | 27,521 | 25.14% | 15,120 | 19.62% |
| 25,000-49,999 | 11,366 | 22.72% | 16,166 | 25.53% | 28,469 | 26.01% | 18,736 | 24.31% |
| 50,000-74,999 | 9,084 | 18.16% | 12,995 | 20.52% | 19,511 | 17.82% | 14,661 | 19.02% |
| 75,000-99,999 | 6,517 | 13.03% | 8,094 | 12.78% | 11,894 | 10.86% | 9,779 | 12.69% |
| 100,000-149,999 | 6,966 | 13.93% | 7,978 | 12.60% | 11,937 | 10.90% | 10,331 | 13.41% |
| ≥150,000 | 6,529 | 13.05% | 5,473 | 8.64% | 10,141 | 9.26% | 8,438 | 10.95% |
| Marital Status | | | | | | | | |
| Not Married | 23,732 | 47.45% | 28,888 | 45.62% | 52,779 | 48.21% | 35,002 | 45.42% |
| Married | 26,284 | 52.55% | 34,437 | 54.38% | 56,694 | 51.79% | 42,063 | 54.58% |
| Employment Status | | | | | | | | |
| Not Working | 20,091 | 40.17% | 24,063 | 38.00% | 47,231 | 43.14% | 31,024 | 40.26% |
| Working | 29,925 | 59.83% | 39,262 | 62.00% | 62,242 | 56.86% | 46,041 | 59.74% |

* A total of 1,512 respondents (847 during 2014-15 and 665 during 2018-19) were excluded from analysis due to indeterminate smoking status.

# Appendix Table 3. Changes in Regional and National Prevalence of Current Cigarette Use by Demographic Group, U.S. Adults (18+) – TUS-CPS 2014-15 and 2018-19

| Measure | Year | Region | | | | National |
| --- | --- | --- | --- | --- | --- | --- |
|  |  | Northeast | Midwest | South | West |  |
| **Overall** | **2014-15 (%; 95% CI)** | **12.7 (12.3, 13.2)** | **16.2 (15.8, 16.7)** | **14.9 (14.6, 15.3)** | **10.3 (10, 10.7)** | **13.7 (13.5, 13.9)** |
|  | **2018-19 (%; 95% CI)** | **10.2 (9.8, 10.7)** | **14 (13.5, 14.5)** | **12.1 (11.8, 12.4)** | **9 (8.6, 9.4)** | **11.4 (11.2, 11.6)** |
|  | **Percentage Point Difference (95% CI)** | **-2.5 (-3.2, -1.8)***** | **-2.2 (-2.9, -1.6)***** | **-2.9 (-3.3, -2.4)***** | **-1.3 (-1.9, -0.8)***** | **-2.3 (-2.6, -2)***** |
|  | **Relative Difference** | **-19.8***** | **-13.7***** | **-19.1***** | **-13***** | **-16.9***** |
| Sex | | | | | | |
| Male | 2014-15 (%; 95% CI) | 14.1 (13.4, 14.9) | 17.6 (17, 18.3) | 16.8 (16.3, 17.4) | 12 (11.4, 12.6) | 15.4 (15.1, 15.7) |
|  | 2018-19 (%; 95% CI) | 10.8 (10.1, 11.6) | 15.3 (14.5, 16.1) | 14 (13.5, 14.6) | 10.8 (10.2, 11.4) | 12.9 (12.6, 13.3) |
|  | Percentage Point Difference (95% CI) | -3.3*** (-4.4, -2.2) | -2.4*** (-3.4, -1.3) | -2.8*** (-3.6, -2) | -1.2** (-2.1, -0.4) | -2.4*** (-2.9, -2) |
|  | Relative Difference (%) | -23.4*** | -13.3*** | -16.7*** | -10.4** | -15.8*** |
| Female | 2014-15 (%; 95% CI) | 11.5 (10.9, 12.1) | 14.9 (14.3, 15.4) | 13.2 (12.8, 13.7) | 8.7 (8.3, 9.2) | 12.2 (12, 12.5) |
|  | 2018-19 (%; 95% CI) | 9.7 (9.1, 10.3) | 12.8 (12.1, 13.4) | 10.3 (9.9, 10.7) | 7.3 (6.8, 7.7) | 10 (9.7, 10.2) |
|  | Percentage Point Difference (95% CI) | -1.8*** (-2.7, -0.9) | -2.1*** (-3, -1.2) | -2.9*** (-3.5, -2.3) | -1.4*** (-2.1, -0.8) | -2.2*** (-2.6, -1.9) |
|  | Relative Difference (%) | -15.8*** | -14.1*** | -22*** | -16.6*** | -18.2*** |
| Age Category, Years | | | | | | |
| 18–24 | 2014-15 (%; 95% CI) | 11.5 (9.7, 13.5) | 15.9 (14.3, 17.7) | 13.8 (12.7, 15.1) | 10.2 (9, 11.6) | 13 (12.3, 13.7) |
|  | 2018-19 (%; 95% CI) | 5.5 (4.2, 7.3) | 9.2 (7.6, 11) | 7.7 (6.7, 8.9) | 6.7 (5.5, 8.1) | 7.4 (6.8, 8.1) |
|  | Percentage Point Difference (95% CI) | -5.9*** (-8.4, -3.5) | -6.7*** (-9.1, -4.3) | -6.1*** (-7.7, -4.5) | -3.5*** (-5.4, -1.7) | -5.6*** (-6.6, -4.6) |
|  | Relative Difference (%) | -51.7*** | -42.2*** | -44.1*** | -34.6*** | -42.9*** |
| 25–34 | 2014-15 (%; 95% CI) | 14.9 (13.6, 16.2) | 18.3 (17.2, 19.5) | 17.4 (16.6, 18.3) | 11.8 (10.9, 12.7) | 15.8 (15.3, 16.3) |
|  | 2018-19 (%; 95% CI) | 11.6 (10.3, 12.9) | 15.1 (13.9, 16.5) | 13 (12.2, 13.9) | 8.9 (8, 9.8) | 12.2 (11.6, 12.7) |
|  | Percentage Point Difference (95% CI) | -3.3*** (-5.1, -1.5) | -3.2*** (-4.9, -1.5) | -4.4*** (-5.7, -3.2) | -2.9*** (-4.2, -1.6) | -3.6*** (-4.4, -2.9) |
|  | Relative Difference (%) | -22.2*** | -17.4*** | -25.5*** | -24.6*** | -23.1*** |
| 35–44 | 2014-15 (%; 95% CI) | 15.1 (13.8, 16.4) | 19.3 (18.2, 20.5) | 15.3 (14.5, 16.2) | 10.7 (9.9, 11.6) | 14.9 (14.4, 15.4) |
|  | 2018-19 (%; 95% CI) | 10.8 (9.6, 12) | 16.9 (15.6, 18.2) | 13.9 (13.1, 14.8) | 9.7 (8.9, 10.6) | 12.9 (12.4, 13.4) |
|  | Percentage Point Difference (95% CI) | -4.3*** (-6.1, -2.5) | -2.5** (-4.2, -0.7) | -1.4* (-2.6, -0.2) | -1 (-2.2, 0.2) | -2*** (-2.7, -1.3) |
|  | Relative Difference (%) | -28.5*** | -12.8** | -9.1* | -9.1 | -13.5*** |
| 45-64 | 2014-15 (%; 95% CI) | 14.7 (13.9, 15.5) | 18.4 (17.7, 19.2) | 17.3 (16.8, 17.9) | 11.6 (11, 12.2) | 15.8 (15.4, 16.1) |
|  | 2018-19 (%; 95% CI) | 13.3 (12.4, 14.2) | 17.3 (16.5, 18.2) | 15.1 (14.5, 15.7) | 11.1 (10.4, 11.8) | 14.3 (13.9, 14.7) |
|  | Percentage Point Difference (95% CI) | -1.4* (-2.6, -0.2) | -1.1 (-2.3, 0) | -2.3*** (-3.1, -1.5) | -0.5 (-1.4, 0.4) | -1.5*** (-2, -1) |
|  | Relative Difference (%) | -9.7* | -6 | -13.2*** | -4.2 | -9.4*** |
| 65+ | 2014-15 (%; 95% CI) | 6.2 (5.6, 6.9) | 8.1 (7.5, 8.8) | 8.7 (8.2, 9.2) | 6.1 (5.5, 6.7) | 7.5 (7.2, 7.8) |
|  | 2018-19 (%; 95% CI) | 6.3 (5.7, 7.1) | 8.1 (7.4, 8.8) | 7.8 (7.3, 8.3) | 6.3 (5.7, 6.9) | 7.2 (6.9, 7.5) |
|  | Percentage Point Difference (95% CI) | 0.1 (-0.9, 1.1) | -0.1 (-1, 0.9) | -0.9* (-1.6, -0.2) | 0.2 (-0.6, 1) | -0.3 (-0.7, 0.1) |
|  | Relative Difference (%) | 1.6 | -0.9 | -10.4* | 3.2 | -3.8 |
| Race/Ethnicity | | | | | | |
| White, non-Hispanic | 2014-15 (%; 95% CI) | 13.4 (12.9, 14) | 16.5 (16, 17) | 16.9 (16.5, 17.4) | 11.4 (11, 11.9) | 15 (14.8, 15.3) |
|  | 2018-19 (%; 95% CI) | 11.2 (10.6, 11.8) | 14.3 (13.8, 14.8) | 13.5 (13.1, 14) | 10 (9.5, 10.5) | 12.6 (12.3, 12.8) |
|  | Percentage Point Difference (95% CI) | -2.2*** (-3, -1.4) | -2.2*** (-2.9, -1.5) | -3.4*** (-4, -2.8) | -1.5*** (-2.1, -0.8) | -2.5*** (-2.8, -2.1) |
|  | Relative Difference (%) | -16.6*** | -13.4*** | -19.9*** | -12.8*** | -16.4*** |
| Black, non-Hispanic | 2014-15 (%; 95% CI) | 14 (12.4, 15.8) | 17.2 (15.6, 18.9) | 14.3 (13.5, 15.1) | 13.4 (11.4, 15.7) | 14.7 (14, 15.3) |
|  | 2018-19 (%; 95% CI) | 11.4 (9.7, 13.3) | 16.3 (14.4, 18.4) | 12.1 (11.3, 12.9) | 10.9 (8.8, 13.4) | 12.6 (11.9, 13.3) |
|  | Percentage Point Difference (95% CI) | -2.7* (-5.1, -0.2) | -0.9 (-3.5, 1.7) | -2.2*** (-3.4, -1.1) | -2.5 (-5.6, 0.6) | -2.1*** (-3, -1.2) |
|  | Relative Difference (%) | -19* | -5.2 | -15.6*** | -18.7 | -14.3*** |
| Hispanic | 2014-15 (%; 95% CI) | 10.4 (9.1, 12) | 11.7 (10, 13.5) | 9.1 (8.3, 9.9) | 8 (7.3, 8.8) | 9.1 (8.6, 9.6) |
|  | 2018-19 (%; 95% CI) | 7 (5.8, 8.4) | 8.5 (6.9, 10.4) | 7.5 (6.8, 8.4) | 7.4 (6.7, 8.2) | 7.5 (7, 8) |
|  | Percentage Point Difference (95% CI) | -3.5*** (-5.4, -1.5) | -3.2* (-5.7, -0.7) | -1.6** (-2.7, -0.4) | -0.6 (-1.7, 0.5) | -1.6*** (-2.3, -0.9) |
|  | Relative Difference (%) | -33.2*** | -27.3* | -17.1** | -7.6 | -17.7*** |
| Asian, non-Hispanic | 2014-15 (%; 95% CI) | 6.9 (5.4, 8.8) | 8.9 (6.9, 11.5) | 6.2 (5, 7.7) | 6.2 (5.2, 7.3) | 6.7 (6, 7.4) |
|  | 2018-19 (%; 95% CI) | 4.2 (3.1, 5.7) | 5.4 (3.8, 7.8) | 5.5 (4.3, 7) | 5.2 (4.3, 6.3) | 5.1 (4.5, 5.8) |
|  | Percentage Point Difference (95% CI) | -2.7* (-4.8, -0.6) | -3.5* (-6.5, -0.5) | -0.7 (-2.6, 1.2) | -1 (-2.4, 0.4) | -1.5** (-2.5, -0.6) |
|  | Relative Difference (%) | -38.8* | -39.2* | -11.2 | -15.5 | -23.3** |
| Other, non-Hispanic | 2014-15 (%; 95% CI) | 18.7 (13.5, 25.3) | 26 (22, 30.4) | 22.4 (19.3, 25.7) | 17.4 (15.2, 19.9) | 20.6 (19, 22.4) |
|  | 2018-19 (%; 95% CI) | 15.5 (11, 21.4) | 23.3 (18.6, 28.8) | 19.1 (16.3, 22.3) | 15 (12.8, 17.4) | 17.8 (16.2, 19.5) |
|  | Percentage Point Difference (95% CI) | -3.2 (-11, 4.6) | -2.7 (-9.3, 3.9) | -3.3 (-7.6, 1.1) | -2.4 (-5.7, 0.8) | -2.8* (-5.2, -0.5) |
|  | Relative Difference (%) | -17.2 | -10.3 | -14.5 | -14.1 | -13.8* |
| Education | | | | | | |
| 12th Grade or below (No Diploma) | 2014-15 (%; 95% CI) | 19 (17.2, 21) | 28.1 (26.2, 30.1) | 23.4 (22.2, 24.5) | 12.3 (11.2, 13.6) | 20.7 (20, 21.4) |
|  | 2018-19 (%; 95% CI) | 16.7 (14.7, 19) | 25 (22.6, 27.5) | 19.9 (18.6, 21.2) | 12.1 (10.7, 13.7) | 18.2 (17.4, 19.1) |
|  | Percentage Point Difference (95% CI) | -2.3 (-5.2, 0.5) | -3.1 (-6.3, 0) | -3.5*** (-5.2, -1.8) | -0.2 (-2.1, 1.7) | -2.5*** (-3.6, -1.4) |
|  | Relative Difference (%) | -12.2 | -11.1 | -14.9*** | -1.8 | -12*** |
| Graduation from high school | 2014-15 (%; 95% CI) | 17.5 (16.5, 18.7) | 20.3 (19.3, 21.2) | 17.5 (16.7, 18.2) | 14 (13.1, 14.9) | 17.4 (17, 17.9) |
|  | 2018-19 (%; 95% CI) | 13.9 (12.9, 15) | 18.7 (17.6, 19.8) | 16.2 (15.4, 17) | 11.8 (10.9, 12.8) | 15.4 (14.9, 15.9) |
|  | Percentage Point Difference (95% CI) | -3.7*** (-5.2, -2.1) | -1.6* (-3.1, -0.2) | -1.3* (-2.3, -0.2) | -2.2** (-3.5, -0.9) | -2*** (-2.7, -1.4) |
|  | Relative Difference (%) | -20.8*** | -7.9* | -7.4* | -15.7** | -11.7*** |
| GED or other equivalent | 2014-15 (%; 95% CI) | 38.6 (34.1, 43.4) | 40 (36.4, 43.7) | 36.4 (34, 38.9) | 28.1 (24.9, 31.5) | 35.8 (34.2, 37.5) |
|  | 2018-19 (%; 95% CI) | 30.6 (25.8, 36) | 34.9 (30.9, 39.2) | 29.1 (26.5, 31.9) | 26.3 (22.7, 30.3) | 30 (28.2, 31.8) |
|  | Percentage Point Difference (95% CI) | -8* (-14.9, -1) | -5 (-10.6, 0.5) | -7.3*** (-10.9, -3.7) | -1.8 (-6.8, 3.3) | -5.9*** (-8.3, -3.4) |
|  | Relative Difference (%) | -20.6* | -12.6 | -20*** | -6.3 | -16.4*** |
| Some College or Above | 2014-15 (%; 95% CI) | 8.8 (8.3, 9.3) | 11.7 (11.2, 12.2) | 10.7 (10.3, 11.1) | 8.1 (7.7, 8.5) | 9.9 (9.7, 10.1) |
|  | 2018-19 (%; 95% CI) | 7.3 (6.8, 7.8) | 10.1 (9.6, 10.6) | 8.3 (7.9, 8.6) | 7 (6.6, 7.4) | 8.2 (8, 8.4) |
|  | Percentage Point Difference (95% CI) | -1.5*** (-2.2, -0.7) | -1.6*** (-2.3, -0.9) | -2.4*** (-2.9, -1.9) | -1.1*** (-1.6, -0.5) | -1.7*** (-2, -1.4) |
|  | Relative Difference (%) | -16.8*** | -13.6*** | -22.6*** | -13.1*** | -17.5*** |
| Marital Status | | | | | | |
| Not Married | 2014-15 (%; 95% CI) | 16.4 (15.6, 17.2) | 21 (20.2, 21.7) | 18.8 (18.3, 19.4) | 13.8 (13.2, 14.4) | 17.7 (17.3, 18) |
|  | 2018-19 (%; 95% CI) | 13.2 (12.5, 14) | 18.1 (17.3, 18.9) | 14.9 (14.4, 15.4) | 11.5 (10.9, 12.1) | 14.4 (14.1, 14.8) |
|  | Percentage Point Difference (95% CI) | -3.1*** (-4.2, -2) | -2.9*** (-4, -1.8) | -4*** (-4.7, -3.2) | -2.3*** (-3.2, -1.5) | -3.2*** (-3.7, -2.8) |
|  | Relative Difference (%) | -19.2*** | -13.8*** | -21*** | -16.8*** | -18.3*** |
| Married | 2014-15 (%; 95% CI) | 8.9 (8.4, 9.5) | 11.6 (11.1, 12.2) | 11.1 (10.7, 11.5) | 7 (6.7, 7.4) | 9.9 (9.6, 10.1) |
|  | 2018-19 (%; 95% CI) | 7 (6.5, 7.6) | 10 (9.5, 10.6) | 9.3 (8.9, 9.7) | 6.5 (6.1, 7) | 8.4 (8.1, 8.6) |
|  | Percentage Point Difference (95% CI) | -1.9*** (-2.7, -1.1) | -1.6*** (-2.4, -0.9) | -1.9*** (-2.4, -1.3) | -0.5 (-1.1, 0.1) | -1.5*** (-1.8, -1.2) |
|  | Relative Difference (%) | -21.3*** | -14.2*** | -16.7*** | -7.3 | -15.2*** |
| Employment Status | | | | | | |
| Not Working | 2014-15 (%; 95% CI) | 13.5 (12.7, 14.3) | 16.7 (15.9, 17.4) | 16.6 (16, 17.1) | 10.8 (10.2, 11.3) | 14.7 (14.4, 15) |
|  | 2018-19 (%; 95% CI) | 11.2 (10.4, 12) | 15.1 (14.3, 16) | 13.4 (12.9, 13.9) | 9.5 (8.9, 10.1) | 12.4 (12.1, 12.8) |
|  | Percentage Point Difference (95% CI) | -2.3*** (-3.5, -1.2) | -1.5** (-2.6, -0.4) | -3.2*** (-3.9, -2.4) | -1.2** (-2.1, -0.4) | -2.3*** (-2.7, -1.8) |
|  | Relative Difference (%) | -17.3*** | -9.2** | -19.1*** | -11.5** | -15.4*** |
| Working | 2014-15 (%; 95% CI) | 12.3 (11.7, 12.9) | 15.9 (15.4, 16.5) | 13.8 (13.4, 14.3) | 10 (9.6, 10.5) | 13.1 (12.9, 13.4) |
|  | 2018-19 (%; 95% CI) | 9.7 (9.1, 10.3) | 13.4 (12.8, 14) | 11.3 (10.9, 11.7) | 8.7 (8.2, 9.1) | 10.8 (10.6, 11.1) |
|  | Percentage Point Difference (95% CI) | -2.6*** (-3.5, -1.7) | -2.6*** (-3.4, -1.8) | -2.6*** (-3.2, -2) | -1.4*** (-2, -0.7) | -2.3*** (-2.7, -1.9) |
|  | Relative Difference (%) | -21.2*** | -16.2*** | -18.6*** | -13.8*** | -17.6*** |
| Annual Household Income, $USD | | | | | | |
| Below $25,000 | 2014-15 (%; 95% CI) | 19.7 (18.4, 21) | 25.3 (24.2, 26.5) | 22.6 (21.9, 23.4) | 16.2 (15.3, 17.2) | 21.3 (20.8, 21.8) |
|  | 2018-19 (%; 95% CI) | 16.3 (15, 17.8) | 23.4 (22, 24.9) | 18.8 (17.9, 19.6) | 14.8 (13.7, 16) | 18.4 (17.9, 19) |
|  | Percentage Point Difference (95% CI) | -3.4*** (-5.3, -1.5) | -1.9* (-3.8, 0) | -3.9*** (-5, -2.8) | -1.4 (-2.8, 0.1) | -2.8*** (-3.6, -2.1) |
|  | Relative Difference (%) | -17.1*** | -7.5* | -17.2*** | -8.4 | -13.4*** |
| $25,000-$49,999 | 2014-15 (%; 95% CI) | 14.9 (13.8, 16) | 18.2 (17.3, 19.1) | 16.2 (15.5, 16.9) | 12 (11.3, 12.8) | 15.5 (15.1, 15.9) |
|  | 2018-19 (%; 95% CI) | 12.9 (11.7, 14.1) | 17.2 (16.1, 18.3) | 13.8 (13.1, 14.6) | 10.4 (9.6, 11.3) | 13.6 (13.1, 14.1) |
|  | Percentage Point Difference (95% CI) | -2* (-3.6, -0.5) | -1 (-2.4, 0.4) | -2.4*** (-3.4, -1.4) | -1.6** (-2.7, -0.4) | -1.9*** (-2.5, -1.3) |
|  | Relative Difference (%) | -13.7* | -5.5 | -14.7*** | -13.2** | -12.2*** |
| $50,000-$74,999 | 2014-15 (%; 95% CI) | 12.6 (11.5, 13.7) | 14.6 (13.7, 15.6) | 12.2 (11.5, 13) | 9.5 (8.7, 10.3) | 12.2 (11.8, 12.7) |
|  | 2018-19 (%; 95% CI) | 11.6 (10.4, 12.9) | 14.1 (13, 15.2) | 12.4 (11.6, 13.2) | 9.6 (8.8, 10.6) | 12 (11.5, 12.5) |
|  | Percentage Point Difference (95% CI) | -1 (-2.7, 0.7) | -0.5 (-1.9, 0.9) | 0.2 (-0.9, 1.3) | 0.2 (-1, 1.4) | -0.2 (-0.9, 0.4) |
|  | Relative Difference (%) | -8 | -3.5 | 1.4 | 1.9 | -1.9 |
| $75,000-$99,999 | 2014-15 (%; 95% CI) | 10.4 (9.2, 11.7) | 11.5 (10.5, 12.6) | 10.1 (9.3, 11) | 7.3 (6.5, 8.2) | 9.8 (9.3, 10.3) |
|  | 2018-19 (%; 95% CI) | 8.5 (7.3, 9.8) | 10.8 (9.7, 12) | 8.3 (7.5, 9.2) | 7.8 (6.9, 8.9) | 8.8 (8.3, 9.3) |
|  | Percentage Point Difference (95% CI) | -1.9* (-3.6, -0.1) | -0.7 (-2.3, 0.9) | -1.8** (-3, -0.6) | 0.5 (-0.8, 1.8) | -1** (-1.7, -0.3) |
|  | Relative Difference (%) | -18.3* | -6.1 | -17.7** | 7 | -10.5** |
| $100,000-$149,999 | 2014-15 (%; 95% CI) | 6.8 (5.9, 7.9) | 9.5 (8.5, 10.6) | 7.7 (6.9, 8.5) | 5.8 (5, 6.7) | 7.4 (7, 7.9) |
|  | 2018-19 (%; 95% CI) | 7.1 (6.1, 8.2) | 7.1 (6.2, 8.1) | 7 (6.3, 7.8) | 5.8 (5, 6.7) | 6.7 (6.3, 7.2) |
|  | Percentage Point Difference (95% CI) | 0.2 (-1.2, 1.7) | -2.4*** (-3.8, -1) | -0.7 (-1.8, 0.4) | 0 (-1.2, 1.1) | -0.7* (-1.3, 0) |
|  | Relative Difference (%) | 3.3 | -25*** | -8.9 | -0.5 | -9.1* |
| $150,000+ | 2014-15 (%; 95% CI) | 5.8 (4.8, 6.8) | 4.1 (3.4, 5) | 5.3 (4.6, 6.1) | 4.3 (3.6, 5.1) | 4.9 (4.5, 5.4) |
|  | 2018-19 (%; 95% CI) | 3.9 (3.2, 4.7) | 5.4 (4.5, 6.5) | 4.7 (4, 5.5) | 3.7 (3.1, 4.5) | 4.4 (4, 4.8) |
|  | Percentage Point Difference (95% CI) | -1.9** (-3.2, -0.6) | 1.3* (0, 2.6) | -0.6 (-1.7, 0.5) | -0.5 (-1.6, 0.5) | -0.5 (-1.1, 0) |
|  | Relative Difference (%) | -32.9** | 30.8* | -11.4 | -12.3 | -11 |

We indicate the level of significance for differences between survey years as follows: ^***^p<0.001, ^**^p<0.01, and ^*^p<0.05. The relative difference between survey years is calculated as: $\frac{Prevalence Estimate [2018-19] - Prevalence Estimate [2014-15]}{Prevalence Estimate [2014-15]}$.

# Appendix Table 4. Changes in Regional and National Quit Interest by Demographic Group, US Adults (18+) – TUS-CPS 2014-15 and 2018-19

| Measure | Year | Region | | | | National |
| --- | --- | --- | --- | --- | --- | --- |
|  |  | Northeast | Midwest | South | West |  |
| **Overall** | **2014-15 (%; 95% CI)** | **80 (78.3, 81.6)** | **77.9 (76.6, 79.2)** | **76.3 (75.3, 77.4)** | **77.3 (75.8, 78.9)** | **77.5** **(76.9, 78.2)** |
|  | **2018-19 (%; 95% CI)** | **79.9 (77.8, 81.8)** | **74.8 (73.1, 76.4)** | **75 (73.7, 76.2)** | **79.6 (77.8, 81.3)** | **76.6 (75.8, 77.4)** |
|  | **Percentage Point Difference (95% CI)** | **0 (-0.03, 0.02)** | **-0.03 (‑0.05, ‑0.01)**** | **-0.01 (-0.03, 0)** | **0.02 (0, 0.05)** | **-0.01 (-0.02, 0)** |
|  | **Relative Difference (%)** | **-0.2** | **-4**** | **-1.8** | **3** | **-1.2** |
| Sex | | | | | | |
| Male | 2014-15 (%; 95% CI) | 79 (76.5, 81.3) | 76.8 (74.9, 78.6) | 75.3 (73.8, 76.8) | 78 (75.8, 80) | 76.8 (75.8, 77.7) |
|  | 2018-19 (%; 95% CI) | 79.4 (76.4, 82.2) | 73.5 (71, 75.8) | 73.7 (71.9, 75.5) | 78.9 (76.3, 81.2) | 75.5 (74.4, 76.6) |
|  | Percentage Point Difference (95% CI) | 0.4 (-3.3, 4.2) | -3.4* (-6.4, -0.4) | -1.6 (-3.9, 0.8) | 0.9 (-2.3, 4.1) | -1.3 (-2.7, 0.2) |
|  | Relative Difference (%) | 0.6 | -4.4* | -2.1 | 1.1 | -1.6 |
| Female | 2014-15 (%; 95% CI) | 81.2 (78.8, 83.3) | 79.1 (77.3, 80.8) | 77.6 (76.1, 79) | 76.5 (74.2, 78.7) | 78.4 (77.5, 79.3) |
|  | 2018-19 (%; 95% CI) | 80.3 (77.4, 82.9) | 76.3 (74, 78.4) | 76.6 (74.8, 78.2) | 80.7 (78.2, 83) | 77.8 (76.7, 78.9) |
|  | Percentage Point Difference (95% CI) | -0.9 (-4.4, 2.6) | -2.8* (-5.7, 0) | -1 (-3.3, 1.2) | 4.2* (0.9, 7.5) | -0.6 (-2, 0.9) |
|  | Relative Difference (%) | -1.1 | -3.6* | -1.3 | 5.4* | -0.7 |
| Age Category, Years | | | | | | |
| 18–24 | 2014-15 (%; 95% CI) | 84.6 (76.9, 90.1) | 81.2 (76.3, 85.3) | 78.6 (74.5, 82.2) | 80.5 (74.9, 85.1) | 80.5 (78, 82.7) |
|  | 2018-19 (%; 95% CI) | 78.4 (65.1, 87.6) | 85.3 (77.8, 90.6) | 75.8 (69.3, 81.3) | 89 (82.7, 93.2) | 81.5 (77.7, 84.7) |
|  | Percentage Point Difference (95% CI) | -6.2 (-19.2, 6.9) | 4.1 (-3.7, 11.9) | -2.8 (-10, 4.3) | 8.5* (1.3, 15.7) | 1 (-3.2, 5.2) |
|  | Relative Difference (%) | -7.3 | 5.1 | -3.6 | 10.6* | 1.2 |
| 25–34 | 2014-15 (%; 95% CI) | 83.1 (79.3, 86.3) | 80.3 (77.3, 82.9) | 80.2 (77.9, 82.4) | 82 (78.5, 85) | 81 (79.6, 82.4) |
|  | 2018-19 (%; 95% CI) | 85.1 (80.4, 88.8) | 77.9 (73.8, 81.5) | 77.3 (74.1, 80.1) | 82.6 (78, 86.4) | 79.6 (77.7, 81.4) |
|  | Percentage Point Difference (95% CI) | 2 (-3.4, 7.4) | -2.3 (-7.1, 2.4) | -3 (-6.7, 0.8) | 0.6 (-4.7, 5.9) | -1.4 (-3.7, 0.9) |
|  | Relative Difference (%) | 2.4 | -2.9 | -3.7 | 0.7 | -1.7 |
| 35–44 | 2014-15 (%; 95% CI) | 84 (80.3, 87.1) | 80.4 (77.5, 83) | 77.8 (75.3, 80.1) | 82.7 (79.2, 85.8) | 80.5 (79, 81.9) |
|  | 2018-19 (%; 95% CI) | 78 (72.5, 82.6) | 73.8 (69.8, 77.4) | 75.6 (72.7, 78.3) | 81.4 (77.5, 84.8) | 76.6 (74.7, 78.4) |
|  | Percentage Point Difference (95% CI) | -6 (-12.1, 0) | -6.6** (-11.3, -1.9) | -2.1 (-5.9, 1.6) | -1.3 (-6.2, 3.6) | -3.9** (-6.2, -1.6) |
|  | Relative Difference (%) | -7.2 | -8.2** | -2.8 | -1.6 | -4.8** |
| 45-64 | 2014-15 (%; 95% CI) | 79 (76.4, 81.4) | 76.3 (74.3, 78.3) | 75.6 (74, 77.1) | 75.2 (72.7, 77.5) | 76.3 (75.3, 77.3) |
|  | 2018-19 (%; 95% CI) | 81.1 (78, 83.8) | 74.1 (71.6, 76.4) | 76.5 (74.7, 78.3) | 78.3 (75.3, 80.9) | 77 (75.8, 78.1) |
|  | Percentage Point Difference (95% CI) | 2 (-1.8, 5.8) | -2.3 (-5.4, 0.8) | 0.9 (-1.5, 3.4) | 3.1 (-0.6, 6.8) | 0.7 (-0.9, 2.2) |
|  | Relative Difference (%) | 2.6 | -3 | 1.2 | 4.1 | 0.9 |
| 65+ | 2014-15 (%; 95% CI) | 63.9 (58.2, 69.3) | 70.7 (66.7, 74.4) | 66.7 (63.5, 69.7) | 62 (56.8, 66.8) | 66.4 (64.3, 68.4) |
|  | 2018-19 (%; 95% CI) | 71.2 (65.7, 76.1) | 67.4 (63, 71.5) | 65.5 (62.2, 68.7) | 70.2 (65.4, 74.6) | 67.7 (65.6, 69.8) |
|  | Percentage Point Difference (95% CI) | 7.3 (-0.3, 14.9) | -3.3 (-9, 2.4) | -1.2 (-5.7, 3.3) | 8.2* (1.4, 15) | 1.3 (-1.6, 4.3) |
|  | Relative Difference (%) | 11.4 | -4.7 | -1.7 | 13.3* | 2 |
| Race/Ethnicity | | | | | | |
| White, non-Hispanic | 2014-15 (%; 95% CI) | 79.2 (77.3, 81) | 77.8 (76.4, 79.2) | 75.4 (74.1, 76.6) | 77.3 (75.4, 79) | 77 (76.3, 77.8) |
|  | 2018-19 (%; 95% CI) | 79 (76.7, 81.1) | 74.7 (72.9, 76.4) | 73.2 (71.7, 74.7) | 78.7 (76.6, 80.7) | 75.5 (74.6, 76.4) |
|  | Percentage Point Difference (95% CI) | -0.3 (-3.2, 2.6) | -3.1** (-5.4, -0.9) | -2.2* (-4.1, -0.2) | 1.5 (-1.3, 4.2) | -1.5* (-2.7, -0.4) |
|  | Relative Difference (%) | -0.3 | -4** | -2.9* | 1.9 | -2* |
| Black, non-Hispanic | 2014-15 (%; 95% CI) | 82.4 (76.5, 87) | 79.9 (75.4, 83.8) | 80.3 (77.7, 82.7) | 77.4 (69.9, 83.6) | 80.3 (78.3, 82.1) |
|  | 2018-19 (%; 95% CI) | 87 (80.1, 91.8) | 79.2 (73, 84.3) | 77.1 (73.8, 80.1) | 75.6 (64.1, 84.3) | 78.8 (76.3, 81.2) |
|  | Percentage Point Difference (95% CI) | 4.7 (-3.1, 12.4) | -0.7 (-7.7, 6.4) | -3.2 (-7.2, 0.8) | -1.9 (-14.1, 10.4) | -1.5 (-4.6, 1.6) |
|  | Relative Difference (%) | 5.7 | -0.9 | -4 | -2.4 | -1.8 |
| Hispanic | 2014-15 (%; 95% CI) | 80.9 (74.2, 86.2) | 76 (68.4, 82.3) | 74 (69.8, 77.8) | 79.2 (75, 82.9) | 77.1 (74.7, 79.5) |
|  | 2018-19 (%; 95% CI) | 79.6 (71, 86.1) | 67.2 (56, 76.8) | 78.6 (73.7, 82.9) | 82.9 (78.6, 86.5) | 79.3 (76.4, 81.9) |
|  | Percentage Point Difference (95% CI) | -1.3 (-10.9, 8.3) | -8.8 (-21.4, 3.8) | 4.6 (-1.5, 10.7) | 3.7 (-1.9, 9.3) | 2.2 (-1.5, 5.8) |
|  | Relative Difference (%) | -1.6 | -11.6 | 6.2 | 4.7 | 2.8 |
| Asian, non-Hispanic | 2014-15 (%; 95% CI) | 91.6 (80.9, 96.6) | 81.7 (67, 90.8) | 85.1 (75.7, 91.3) | 79.8 (72.5, 85.5) | 83.8 (79.4, 87.4) |
|  | 2018-19 (%; 95% CI) | 75.2 (59.1, 86.4) | 67.4 (48.4, 82) | 79.8 (68.5, 87.8) | 80.7 (72, 87.2) | 78 (72.2, 82.8) |
|  | Percentage Point Difference (95% CI) | -16.4* (-32, -0.8) | -14.3 (-35.3, 6.7) | -5.4 (-17.7, 7) | 1 (-9, 11) | -5.8 (-12.4, 0.8) |
|  | Relative Difference (%) | -17.9* | -17.5 | -6.3 | 1.2 | -6.9 |
| Other, non-Hispanic | 2014-15 (%; 95% CI) | 70.2 (51.1, 84.1) | 75.3 (66, 82.7) | 78.6 (71.6, 84.3) | 70 (62.8, 76.3) | 74.3 (70.1, 78.2) |
|  | 2018-19 (%; 95% CI) | 79.6 (59.9, 91) | 75 (63.6, 83.8) | 84 (77.4, 88.9) | 79.3 (71.7, 85.3) | 80.1 (75.8, 83.8) |
|  | Percentage Point Difference (95% CI) | 9.4 (-13.6, 32.4) | -0.3 (-13.5, 12.9) | 5.3 (-3.2, 13.9) | 9.3 (-0.3, 19) | 5.8* (0.1, 11.5) |
|  | Relative Difference (%) | 13.4 | -0.3 | 6.8 | 13.3 | 7.8* |
| Education | | | | | | |
| 12th Grade or below (No Diploma) | 2014-15 (%; 95% CI) | 78.4 (73.6, 82.5) | 74.8 (71, 78.2) | 70.7 (68.1, 73.2) | 74.3 (69.7, 78.5) | 73.3 (71.5, 75) |
|  | 2018-19 (%; 95% CI) | 76.5 (70.4, 81.6) | 65.1 (59.6, 70.2) | 71 (67.8, 74.1) | 76.9 (71, 81.8) | 71.5 (69.2, 73.7) |
|  | Percentage Point Difference (95% CI) | -1.9 (-9, 5.3) | -9.7** (-16.1, -3.2) | 0.3 (-3.8, 4.4) | 2.5 (-4.4, 9.5) | -1.8 (-4.6, 1.1) |
|  | Relative Difference (%) | -2.4 | -12.9** | 0.4 | 3.4 | -2.4 |
| Graduation from high school | 2014-15 (%; 95% CI) | 77.3 (74.3, 80) | 75.3 (72.9, 77.5) | 74.9 (72.9, 76.9) | 73.6 (70.4, 76.6) | 75.3 (74, 76.5) |
|  | 2018-19 (%; 95% CI) | 79 (75.4, 82.3) | 74.5 (71.6, 77.2) | 73.2 (70.9, 75.5) | 76 (72.2, 79.5) | 75 (73.5, 76.4) |
|  | Percentage Point Difference (95% CI) | 1.7 (-2.7, 6.2) | -0.8 (-4.5, 2.8) | -1.7 (-4.7, 1.3) | 2.4 (-2.3, 7.2) | -0.3 (-2.2, 1.6) |
|  | Relative Difference (%) | 2.3 | -1.1 | -2.3 | 3.3 | -0.4 |
| GED or other equivalent | 2014-15 (%; 95% CI) | 79.8 (72.4, 85.6) | 78.2 (72.9, 82.7) | 75.2 (71.4, 78.7) | 75.9 (70.1, 80.9) | 76.7 (74.2, 79.1) |
|  | 2018-19 (%; 95% CI) | 77.6 (68.5, 84.7) | 70.3 (63.3, 76.5) | 75.4 (70.4, 79.9) | 76 (68.5, 82.2) | 74.6 (71.3, 77.6) |
|  | Percentage Point Difference (95% CI) | -2.2 (-12.7, 8.3) | -7.9 (-16.1, 0.3) | 0.2 (-5.8, 6.2) | 0.1 (-8.7, 8.9) | -2.2 (-6.1, 1.8) |
|  | Relative Difference (%) | -2.8 | -10.1 | 0.3 | 0.2 | -2.8 |
| Some College or Above | 2014-15 (%; 95% CI) | 82.9 (80.4, 85.2) | 80.9 (79.1, 82.6) | 80.3 (78.7, 81.7) | 80.4 (78.3, 82.4) | 80.9 (79.9, 81.8) |
|  | 2018-19 (%; 95% CI) | 81.8 (78.8, 84.5) | 78.4 (76.1, 80.5) | 77.9 (76, 79.7) | 82.7 (80.5, 84.8) | 79.7 (78.6, 80.8) |
|  | Percentage Point Difference (95% CI) | -1.1 (-4.8, 2.7) | -2.5 (-5.3, 0.3) | -2.3 (-4.7, 0.1) | 2.3 (-0.7, 5.2) | -1.2 (-2.6, 0.2) |
|  | Relative Difference (%) | -1.3 | -3.1 | -2.9 | 2.8 | -1.5 |
| Marital Status | | | | | | |
| Not Married | 2014-15 (%; 95% CI) | 79.7 (77.5, 81.8) | 77.7 (76, 79.3) | 76.3 (74.9, 77.6) | 76.5 (74.5, 78.4) | 77.3 (76.4, 78.1) |
|  | 2018-19 (%; 95% CI) | 79.6 (77, 81.9) | 74.5 (72.4, 76.6) | 74.5 (72.9, 76.1) | 80.8 (78.5, 82.8) | 76.5 (75.5, 77.5) |
|  | Percentage Point Difference (95% CI) | -0.1 (-3.4, 3.1) | -3.1* (-5.8, -0.5) | -1.7 (-3.9, 0.4) | 4.3** (1.3, 7.2) | -0.7 (-2.1, 0.6) |
|  | Relative Difference (%) | -0.2 | -4* | -2.3 | 5.6** | -0.9 |
| Married | 2014-15 (%; 95% CI) | 80.6 (77.9, 83.1) | 78.3 (76.2, 80.3) | 76.4 (74.7, 78) | 78.9 (76.3, 81.2) | 78 (76.9, 79) |
|  | 2018-19 (%; 95% CI) | 80.4 (76.8, 83.6) | 75.2 (72.5, 77.8) | 75.7 (73.6, 77.7) | 77.7 (74.6, 80.5) | 76.6 (75.3, 77.9) |
|  | Percentage Point Difference (95% CI) | -0.1 (-4.4, 4.1) | -3.1 (-6.4, 0.2) | -0.7 (-3.4, 1.9) | -1.2 (-5.1, 2.6) | -1.4 (-3, 0.3) |
|  | Relative Difference (%) | -0.2 | -4 | -1 | -1.5 | -1.7 |
| Employment Status | | | | | | |
| Not Working | 2014-15 (%; 95% CI) | 77.8 (75.1, 80.3) | 76.2 (74, 78.2) | 73.1 (71.5, 74.7) | 73.2 (70.5, 75.6) | 74.6 (73.5, 75.6) |
|  | 2018-19 (%; 95% CI) | 77.4 (74.1, 80.4) | 71.7 (68.9, 74.4) | 72.3 (70.3, 74.1) | 75.9 (72.9, 78.7) | 73.6 (72.3, 74.8) |
|  | Percentage Point Difference (95% CI) | -0.4 (-4.5, 3.7) | -4.5* (-7.9, -1) | -0.9 (-3.4, 1.6) | 2.8 (-1.1, 6.6) | -1 (-2.6, 0.7) |
|  | Relative Difference (%) | -0.5 | -5.9* | -1.2 | 3.8 | -1.3 |
| Working | 2014-15 (%; 95% CI) | 81.6 (79.3, 83.6) | 79 (77.3, 80.5) | 78.9 (77.5, 80.3) | 80.1 (78.1, 82) | 79.6 (78.7, 80.4) |
|  | 2018-19 (%; 95% CI) | 81.5 (78.8, 83.9) | 76.7 (74.6, 78.6) | 77 (75.2, 78.6) | 82 (79.8, 84.1) | 78.6 (77.5, 79.6) |
|  | Percentage Point Difference (95% CI) | 0 (-3.4, 3.3) | -2.3 (-4.9, 0.3) | -2 (-4.2, 0.2) | 1.9 (-1, 4.8) | -1 (-2.4, 0.3) |
|  | Relative Difference (%) | -0.1 | -2.9 | -2.5 | 2.4 | -1.3 |
| Annual Household Income, $USD | | | | | | |
| Below $25,000 | 2014-15 (%; 95% CI) | 78.4 (75.3, 81.3) | 77.5 (75.3, 79.6) | 74.6 (72.9, 76.3) | 74.4 (71.6, 77.1) | 75.8 (74.7, 76.9) |
|  | 2018-19 (%; 95% CI) | 75.7 (71.4, 79.5) | 74.3 (71.1, 77.3) | 73.2 (71, 75.3) | 74.4 (70.7, 77.8) | 74 (72.5, 75.5) |
|  | Percentage Point Difference (95% CI) | -2.8 (-7.8, 2.3) | -3.2 (-7, 0.6) | -1.4 (-4.2, 1.3) | 0 (-4.5, 4.5) | -1.8 (-3.6, 0.1) |
|  | Relative Difference (%) | -3.5 | -4.1 | -1.9 | 0 | -2.4 |
| $25,000-$49,999 | 2014-15 (%; 95% CI) | 79 (75.5, 82.1) | 75.7 (73.2, 78) | 76 (74, 77.9) | 77.4 (74.5, 80.1) | 76.6 (75.4, 77.8) |
|  | 2018-19 (%; 95% CI) | 80.7 (76.5, 84.3) | 73.4 (70.3, 76.2) | 74.4 (71.9, 76.7) | 83 (79.9, 85.7) | 76.5 (75, 78) |
|  | Percentage Point Difference (95% CI) | 1.7 (-3.4, 6.8) | -2.3 (-6.2, 1.5) | -1.6 (-4.7, 1.5) | 5.6** (1.6, 9.6) | -0.1 (-2, 1.8) |
|  | Relative Difference (%) | 2.1 | -3.1 | -2.1 | 7.2** | -0.1 |
| $50,000-$74,999 | 2014-15 (%; 95% CI) | 80.9 (76.9, 84.3) | 75.9 (72.7, 78.9) | 79.4 (76.7, 81.8) | 79.9 (76.1, 83.3) | 78.8 (77.2, 80.3) |
|  | 2018-19 (%; 95% CI) | 78.5 (73.5, 82.9) | 75 (71.2, 78.4) | 74.8 (71.5, 77.8) | 79.2 (74.9, 83) | 76.2 (74.3, 78.1) |
|  | Percentage Point Difference (95% CI) | -2.3 (-8.3, 3.7) | -0.9 (-5.7, 3.8) | -4.6* (-8.7, -0.6) | -0.7 (-6.1, 4.7) | -2.5* (-5, -0.1) |
|  | Relative Difference (%) | -2.9 | -1.2 | -5.8* | -0.9 | -3.2* |
| $75,000-$99,999 | 2014-15 (%; 95% CI) | 80.8 (75.6, 85.1) | 87.3 (83.6, 90.3) | 75 (70.9, 78.8) | 83.7 (78.4, 87.9) | 81 (78.8, 83) |
|  | 2018-19 (%; 95% CI) | 84.7 (78.6, 89.2) | 78.5 (73.4, 82.9) | 75.2 (70.2, 79.5) | 82 (76.1, 86.6) | 79.2 (76.6, 81.6) |
|  | Percentage Point Difference (95% CI) | 3.9 (-3.2, 11) | -8.8** (-14.6, -3) | 0.2 (-5.9, 6.2) | -1.8 (-8.8, 5.3) | -1.8 (-5.1, 1.5) |
|  | Relative Difference (%) | 4.8 | -10.1** | 0.2 | -2.1 | -2.2 |
| $100,000-$149,999 | 2014-15 (%; 95% CI) | 81.2 (73.5, 87.1) | 80.4 (75.2, 84.7) | 84.6 (80.4, 87.9) | 79.1 (72.3, 84.6) | 81.7 (79, 84.1) |
|  | 2018-19 (%; 95% CI) | 83.3 (76.9, 88.2) | 75.4 (69.1, 80.8) | 78.1 (73.1, 82.4) | 83.7 (78, 88) | 79.8 (77, 82.3) |
|  | Percentage Point Difference (95% CI) | 2.1 (-6.7, 10.9) | -5 (-12.5, 2.6) | -6.4* (-12.4, -0.5) | 4.6 (-3.3, 12.5) | -1.9 (-5.6, 1.8) |
|  | Relative Difference (%) | 2.6 | -6.2 | -7.6* | 5.8 | -2.3 |
| $150,000+ | 2014-15 (%; 95% CI) | 88.6 (81.6, 93.1) | 87.8 (79.5, 93) | 78.4 (70.8, 84.4) | 75.4 (66.1, 82.8) | 81.9 (78, 85.3) |
|  | 2018-19 (%; 95% CI) | 83.9 (74.9, 90.1) | 76.9 (67.7, 84.2) | 88.3 (82.6, 92.2) | 78.7 (69.4, 85.7) | 82.7 (78.9, 85.9) |
|  | Percentage Point Difference (95% CI) | -4.7 (-14, 4.7) | -10.9* (-21.4, -0.3) | 9.9* (1.6, 18.2) | 3.3 (-8.4, 15) | 0.7 (-4.3, 5.7) |
|  | Relative Difference (%) | -5.3 | -12.4* | 12.6* | 4.3 | 0.9 |

We indicate the level of significance for differences between survey years as follows: ^***^p<0.001, ^**^p<0.01, and ^*^p<0.05. The relative difference between survey years is calculated as: $\frac{Prevalence Estimate [2018-19] - Prevalence Estimate [2014-15]}{Prevalence Estimate [2014-15]}$.

# Appendix Table 5. Changes in Regional and National Doctor’s Advice to Quit by Demographic Group, US Adults (18+) – TUS-CPS 2014-15 and 2018-19

| Measure | Year | Region | | | | National |
| --- | --- | --- | --- | --- | --- | --- |
|  |  | Northeast | Midwest | South | West |  |
| **Overall** | **2014-15 (%; 95% CI)** | **75.4 (73.3, 77.4)** | **73.6 (72, 75.1)** | **69.2 (67.9, 70.6)** | **68.3 (66.2, 70.3)** | **71.4 (70.5, 72.2)** |
|  | **2018-19 (%; 95% CI)** | **74.4 (71.9, 76.9)** | **72.3 (70.3, 74.3)** | **71.9 (70.3, 73.5)** | **68.3 (65.8, 70.7)** | **71.8 (70.8, 72.8)** |
|  | **Percentage Point Difference (95% CI)** | **-1 (-4.2, 2.3)** | **-1.2 (-3.7, 1.3)** | **2.7 (0.6, 4.8)*** | **0 (-3.2, 3.2)** | **0.4 (-0.9, 1.8)** |
|  | **Relative Difference (%)** | **-1.3** | **-1.7** | **3.8*** | **0** | **0.6** |
| Sex | | | | | | |
| Male | 2014-15 (%; 95% CI) | 74.7 (71.4, 77.7) | 72.6 (70.2, 74.9) | 68.4 (66.3, 70.4) | 68.7 (65.7, 71.6) | 70.7 (69.4, 71.9) |
|  | 2018-19 (%; 95% CI) | 74.4 (70.3, 78.1) | 71.2 (68.1, 74.2) | 71 (68.5, 73.4) | 66.9 (63.2, 70.4) | 70.8 (69.2, 72.3) |
|  | Percentage Point Difference (95% CI) | -0.2 (-5.3, 4.8) | -1.4 (-5.2, 2.4) | 2.6 (-0.6, 5.8) | -1.8 (-6.5, 2.8) | 0.1 (-1.9, 2.1) |
|  | Relative Difference (%) | -0.3 | -1.9 | 3.8 | -2.7 | 0.1 |
| Female | 2014-15 (%; 95% CI) | 76.1 (73.3, 78.7) | 74.4 (72.3, 76.4) | 70.1 (68.2, 71.8) | 67.9 (65.1, 70.6) | 72 (70.9, 73.1) |
|  | 2018-19 (%; 95% CI) | 74.5 (71.1, 77.5) | 73.3 (70.7, 75.8) | 72.8 (70.7, 74.8) | 69.9 (66.5, 73.1) | 72.8 (71.4, 74.1) |
|  | Percentage Point Difference (95% CI) | -1.6 (-5.8, 2.5) | -1.1 (-4.4, 2.2) | 2.8* (0, 5.5) | 2 (-2.4, 6.3) | 0.8 (-0.9, 2.5) |
|  | Relative Difference (%) | -2.2 | -1.5 | 3.9* | 2.9 | 1.1 |
| Age Category, Years | | | | | | |
| 18–24 | 2014-15 (%; 95% CI) | 63.7 (53.4, 72.9) | 61.3 (54.2, 67.9) | 55.8 (49.6, 61.7) | 46.4 (37.9, 55.1) | 57.1 (53.3, 60.9) |
|  | 2018-19 (%; 95% CI) | 52.2 (35.9, 68) | 55.2 (44, 65.9) | 59.3 (49.8, 68.1) | 54.7 (41.9, 66.8) | 56.3 (50.4, 62) |
|  | Percentage Point Difference (95% CI) | -11.5 (-30.9, 7.8) | -6.1 (-19.1, 7) | 3.5 (-7.6, 14.6) | 8.3 (-7.2, 23.7) | -0.9 (-7.8, 6.1) |
|  | Relative Difference (%) | -18.1 | -9.9 | 6.3 | 17.8 | -1.5 |
| 25–34 | 2014-15 (%; 95% CI) | 71.4 (66, 76.2) | 68.6 (64.8, 72.2) | 60.2 (56.8, 63.6) | 59.5 (54.3, 64.5) | 64.4 (62.3, 66.5) |
|  | 2018-19 (%; 95% CI) | 73 (66.2, 78.8) | 62.9 (57.5, 67.9) | 61 (56.5, 65.3) | 61.9 (55, 68.4) | 63.7 (61, 66.4) |
|  | Percentage Point Difference (95% CI) | 1.6 (-6.5, 9.7) | -5.7 (-12.1, 0.7) | 0.8 (-4.7, 6.4) | 2.4 (-6, 10.9) | -0.7 (-4.1, 2.8) |
|  | Relative Difference (%) | 2.2 | -8.3 | 1.3 | 4.1 | -1 |
| 35–44 | 2014-15 (%; 95% CI) | 73.3 (68.2, 77.8) | 71.7 (68, 75.1) | 69.8 (66.6, 72.9) | 68.6 (63.8, 73) | 70.8 (68.9, 72.7) |
|  | 2018-19 (%; 95% CI) | 69.3 (62.7, 75.2) | 69.3 (64.4, 73.7) | 70 (66, 73.6) | 62.4 (56.8, 67.7) | 68.2 (65.8, 70.6) |
|  | Percentage Point Difference (95% CI) | -4 (-11.9, 3.9) | -2.4 (-8.3, 3.5) | 0.1 (-4.8, 5) | -6.2 (-13.3, 0.9) | -2.6 (-5.7, 0.5) |
|  | Relative Difference (%) | -5.4 | -3.4 | 0.1 | -9 | -3.7 |
| 45-64 | 2014-15 (%; 95% CI) | 79.2 (76.4, 81.7) | 78.3 (76.1, 80.3) | 74 (72.1, 75.8) | 75.5 (72.7, 78.1) | 76.3 (75.2, 77.4) |
|  | 2018-19 (%; 95% CI) | 77 (73.5, 80.2) | 77.4 (74.8, 79.9) | 76.7 (74.5, 78.7) | 74.3 (70.8, 77.5) | 76.5 (75.1, 77.9) |
|  | Percentage Point Difference (95% CI) | -2.2 (-6.5, 2.1) | -0.8 (-4.1, 2.5) | 2.7 (-0.1, 5.5) | -1.2 (-5.6, 3.1) | 0.2 (-1.5, 2) |
|  | Relative Difference (%) | -2.8 | -1.1 | 3.6 | -1.6 | 0.3 |
| 65+ | 2014-15 (%; 95% CI) | 81.6 (76.9, 85.5) | 78.7 (75.1, 82) | 75.5 (72.5, 78.3) | 75 (70.2, 79.3) | 77.3 (75.4, 79) |
|  | 2018-19 (%; 95% CI) | 81.9 (77, 86) | 81.2 (77.4, 84.5) | 77.6 (74.5, 80.5) | 73.3 (68.2, 77.9) | 78.5 (76.5, 80.3) |
|  | Percentage Point Difference (95% CI) | 0.3 (-5.8, 6.5) | 2.5 (-2.5, 7.5) | 2.1 (-2.1, 6.3) | -1.7 (-8.3, 5) | 1.2 (-1.5, 3.8) |
|  | Relative Difference (%) | 0.4 | 3.2 | 2.8 | -2.2 | 1.5 |
| Race/Ethnicity | | | | | | |
| White, non-Hispanic | 2014-15 (%; 95% CI) | 76.1 (73.8, 78.3) | 73.3 (71.6, 74.9) | 70.5 (68.9, 71.9) | 70 (67.7, 72.3) | 72.3 (71.3, 73.2) |
|  | 2018-19 (%; 95% CI) | 75.3 (72.5, 77.8) | 73.1 (71, 75.1) | 73.4 (71.5, 75.1) | 69.6 (66.7, 72.4) | 73 (71.9, 74.1) |
|  | Percentage Point Difference (95% CI) | -0.8 (-4.3, 2.7) | -0.2 (-2.9, 2.4) | 2.9* (0.6, 5.2) | -0.4 (-4.1, 3.2) | 0.7 (-0.7, 2.2) |
|  | Relative Difference (%) | -1.1 | -0.3 | 4.1* | -0.6 | 1 |
| Black, non-Hispanic | 2014-15 (%; 95% CI) | 75.2 (67.8, 81.3) | 78.4 (73.1, 82.9) | 69.8 (66.3, 73.2) | 69.4 (59.7, 77.6) | 72.6 (70, 75) |
|  | 2018-19 (%; 95% CI) | 75.1 (65, 83) | 71.4 (63.3, 78.3) | 70.9 (66.5, 75) | 73.3 (57.3, 84.9) | 71.8 (68.3, 75.1) |
|  | Percentage Point Difference (95% CI) | -0.1 (-11.4, 11.2) | -7 (-16, 2) | 1.1 (-4.4, 6.6) | 3.9 (-12.8, 20.6) | -0.7 (-5, 3.5) |
|  | Relative Difference (%) | -0.2 | -9 | 1.6 | 5.6 | -1 |
| Hispanic | 2014-15 (%; 95% CI) | 72.9 (64.1, 80.2) | 71.7 (61.5, 80) | 57.2 (50.9, 63.2) | 60.5 (54.4, 66.3) | 63.1 (59.5, 66.6) |
|  | 2018-19 (%; 95% CI) | 70.2 (58.6, 79.7) | 60.4 (46.8, 72.5) | 62.5 (54.9, 69.5) | 59.2 (52.1, 65.9) | 62.2 (57.8, 66.4) |
|  | Percentage Point Difference (95% CI) | -2.7 (-16.1, 10.7) | -11.3 (-27.4, 4.8) | 5.3 (-4.3, 14.9) | -1.3 (-10.5, 7.9) | -0.9 (-6.5, 4.7) |
|  | Relative Difference (%) | -3.7 | -15.8 | 9.3 | -2.2 | -1.4 |
| Asian, non-Hispanic | 2014-15 (%; 95% CI) | 67 (51, 79.9) | 62.6 (44.7, 77.7) | 66.2 (51.1, 78.6) | 70.2 (58.6, 79.7) | 67.4 (60.3, 73.8) |
|  | 2018-19 (%; 95% CI) | 63 (42.4, 79.8) | 77.1 (57.4, 89.3) | 65.9 (51, 78.2) | 79.7 (69.6, 87.1) | 72.9 (65.6, 79.1) |
|  | Percentage Point Difference (95% CI) | -4 (-28.5, 20.6) | 14.4 (-9.1, 37.9) | -0.3 (-20, 19.5) | 9.5 (-4.3, 23.2) | 5.5 (-4.1, 15.1) |
|  | Relative Difference (%) | -6 | 23 | -0.4 | 13.5 | 8.2 |
| Other, non-Hispanic | 2014-15 (%; 95% CI) | 73.6 (49.3, 88.8) | 72.4 (62.1, 80.8) | 68.9 (59.9, 76.8) | 66.6 (57.4, 74.7) | 69.4 (64.1, 74.2) |
|  | 2018-19 (%; 95% CI) | 69.8 (49.7, 84.3) | 67.2 (52.6, 79.1) | 71.5 (62.6, 79) | 68.3 (59, 76.3) | 69.5 (63.9, 74.5) |
|  | Percentage Point Difference (95% CI) | -3.8 (-30.9, 23.4) | -5.3 (-21.7, 11.2) | 2.6 (-9.3, 14.5) | 1.7 (-10.6, 14) | 0.1 (-7.2, 7.4) |
|  | Relative Difference (%) | -5.1 | -7.3 | 3.8 | 2.6 | 0.1 |
| Education | | | | | | |
| 12th Grade or below (No Diploma) | 2014-15 (%; 95% CI) | 81.7 (76.2, 86.1) | 77.7 (73.4, 81.5) | 72.3 (69.2, 75.2) | 66.1 (59.8, 71.8) | 74.4 (72.3, 76.4) |
|  | 2018-19 (%; 95% CI) | 77.7 (69.8, 83.9) | 71.5 (64.5, 77.6) | 75.4 (71.6, 78.9) | 67.2 (58.5, 75) | 73.5 (70.5, 76.2) |
|  | Percentage Point Difference (95% CI) | -4 (-12.6, 4.6) | -6.2 (-13.9, 1.5) | 3.1 (-1.6, 7.8) | 1.2 (-9.1, 11.4) | -0.9 (-4.4, 2.6) |
|  | Relative Difference (%) | -4.9 | -8 | 4.3 | 1.8 | -1.2 |
| Graduation from high school | 2014-15 (%; 95% CI) | 75.2 (71.5, 78.6) | 73.7 (70.9, 76.3) | 70.6 (68, 73.1) | 69.4 (65.3, 73.1) | 72.3 (70.7, 73.7) |
|  | 2018-19 (%; 95% CI) | 74.7 (70.2, 78.7) | 76.1 (72.8, 79.1) | 71.3 (68.2, 74.3) | 68.6 (63.4, 73.5) | 72.9 (71, 74.7) |
|  | Percentage Point Difference (95% CI) | -0.5 (-6, 5) | 2.4 (-1.8, 6.5) | 0.7 (-3.2, 4.6) | -0.7 (-7.1, 5.7) | 0.6 (-1.7, 3) |
|  | Relative Difference (%) | -0.7 | 3.2 | 1 | -1 | 0.9 |
| GED or other equivalent | 2014-15 (%; 95% CI) | 76.3 (67, 83.6) | 80.1 (74.5, 84.8) | 74.4 (69.6, 78.7) | 71.6 (63.5, 78.5) | 75.8 (72.7, 78.6) |
|  | 2018-19 (%; 95% CI) | 75.9 (65.7, 83.9) | 69.3 (60.6, 76.7) | 75.5 (69.8, 80.3) | 71.2 (61.2, 79.5) | 73.2 (69.4, 76.8) |
|  | Percentage Point Difference (95% CI) | -0.3 (-12.7, 12.1) | -10.9* (-20.5, -1.3) | 1.1 (-5.9, 8) | -0.4 (-12.3, 11.6) | -2.5 (-7.3, 2.3) |
|  | Relative Difference (%) | -0.5 | -13.6* | 1.4 | -0.5 | -3.3 |
| Some College or Above | 2014-15 (%; 95% CI) | 73.5 (70.3, 76.5) | 71.5 (69.2, 73.7) | 66.4 (64.3, 68.4) | 67.9 (65.1, 70.6) | 69.3 (68, 70.5) |
|  | 2018-19 (%; 95% CI) | 73.2 (69.3, 76.7) | 70.4 (67.6, 73.2) | 70.7 (68.2, 73) | 68 (64.8, 71) | 70.5 (69, 71.9) |
|  | Percentage Point Difference (95% CI) | -0.3 (-5.2, 4.5) | -1 (-4.6, 2.6) | 4.3** (1.2, 7.4) | 0.1 (-4.1, 4.2) | 1.2 (-0.7, 3.1) |
|  | Relative Difference (%) | -0.5 | -1.4 | 6.4** | 0.1 | 1.7 |
| Marital Status | | | | | | |
| Not Married | 2014-15 (%; 95% CI) | 75.2 (72.4, 77.8) | 72.7 (70.6, 74.7) | 68.8 (67, 70.6) | 66.5 (63.9, 69.1) | 70.7 (69.5, 71.8) |
|  | 2018-19 (%; 95% CI) | 73.8 (70.6, 76.9) | 71.1 (68.3, 73.6) | 70.8 (68.7, 72.9) | 66.5 (63.1, 69.7) | 70.6 (69.3, 72) |
|  | Percentage Point Difference (95% CI) | -1.3 (-5.5, 2.8) | -1.6 (-5, 1.7) | 2 (-0.7, 4.8) | -0.1 (-4.3, 4.2) | 0 (-1.8, 1.7) |
|  | Relative Difference (%) | -1.8 | -2.2 | 3 | -0.1 | 0 |
| Married | 2014-15 (%; 95% CI) | 75.8 (72.6, 78.7) | 74.9 (72.6, 77.2) | 69.9 (67.9, 71.9) | 71.4 (68.1, 74.4) | 72.5 (71.2, 73.7) |
|  | 2018-19 (%; 95% CI) | 75.6 (71.3, 79.5) | 74.3 (71.5, 77) | 73.5 (71.1, 75.9) | 71.3 (67.6, 74.7) | 73.7 (72.1, 75.2) |
|  | Percentage Point Difference (95% CI) | -0.2 (-5.3, 5) | -0.6 (-4.2, 3) | 3.6* (0.5, 6.7) | -0.1 (-4.9, 4.7) | 1.2 (-0.8, 3.2) |
|  | Relative Difference (%) | -0.2 | -0.8 | 5.2* | -0.1 | 1.6 |
| Employment Status | | | | | | |
| Not Working | 2014-15 (%; 95% CI) | 77.8 (74.8, 80.5) | 78 (75.7, 80.2) | 71.7 (69.9, 73.5) | 71.4 (68.5, 74.2) | 74.2 (73.1, 75.4) |
|  | 2018-19 (%; 95% CI) | 77.4 (73.6, 80.7) | 76.8 (73.7, 79.6) | 75.9 (73.8, 77.9) | 70 (66.4, 73.4) | 75.3 (73.9, 76.7) |
|  | Percentage Point Difference (95% CI) | -0.4 (-5, 4.1) | -1.2 (-5, 2.5) | 4.2** (1.4, 6.9) | -1.4 (-5.9, 3.2) | 1.1 (-0.8, 2.9) |
|  | Relative Difference (%) | -0.6 | -1.6 | 5.8** | -1.9 | 1.4 |
| Working | 2014-15 (%; 95% CI) | 73.5 (70.5, 76.3) | 70.5 (68.4, 72.6) | 66.9 (64.9, 68.9) | 65.7 (62.8, 68.5) | 69 (67.8, 70.2) |
|  | 2018-19 (%; 95% CI) | 72.2 (68.6, 75.5) | 69.2 (66.6, 71.7) | 68.5 (66, 70.8) | 66.9 (63.4, 70.3) | 69 (67.6, 70.4) |
|  | Percentage Point Difference (95% CI) | -1.3 (-5.8, 3.2) | -1.3 (-4.6, 2) | 1.5 (-1.5, 4.6) | 1.2 (-3.3, 5.7) | 0 (-1.8, 1.9) |
|  | Relative Difference (%) | -1.8 | -1.9 | 2.3 | 1.8 | 0.1 |
| Annual Household Income, $USD | | | | | | |
| Below $25,000 | 2014-15 (%; 95% CI) | 73.6 (69.5, 77.4) | 75.8 (73.1, 78.3) | 69.6 (67.4, 71.7) | 66.9 (63.2, 70.3) | 71.3 (69.9, 72.7) |
|  | 2018-19 (%; 95% CI) | 77.2 (72.5, 81.3) | 74 (69.9, 77.7) | 73.2 (70.6, 75.7) | 65.2 (60.3, 69.9) | 72.7 (70.9, 74.5) |
|  | Percentage Point Difference (95% CI) | 3.6 (-2.4, 9.5) | -1.8 (-6.5, 2.8) | 3.6* (0.3, 7) | -1.6 (-7.6, 4.4) | 1.4 (-0.9, 3.7) |
|  | Relative Difference (%) | 4.9 | -2.4 | 5.2* | -2.4 | 2 |
| $25,000-$49,999 | 2014-15 (%; 95% CI) | 77.2 (73.1, 80.8) | 73.4 (70.4, 76.2) | 69.2 (66.6, 71.8) | 69.5 (65.6, 73.1) | 71.8 (70.2, 73.4) |
|  | 2018-19 (%; 95% CI) | 72.4 (66.9, 77.3) | 74.2 (70.7, 77.5) | 71.1 (67.8, 74.2) | 66 (60.7, 70.9) | 71.3 (69.3, 73.2) |
|  | Percentage Point Difference (95% CI) | -4.8 (-11.3, 1.7) | 0.8 (-3.6, 5.3) | 1.9 (-2.2, 5.9) | -3.5 (-9.9, 2.8) | -0.5 (-3.1, 2) |
|  | Relative Difference (%) | -6.2 | 1.1 | 2.7 | -5 | -0.7 |
| $50,000-$74,999 | 2014-15 (%; 95% CI) | 77.6 (72.9, 81.7) | 72.8 (69, 76.3) | 69.6 (66.2, 72.8) | 69.2 (64.1, 73.9) | 72 (70, 73.9) |
|  | 2018-19 (%; 95% CI) | 73.1 (66.8, 78.6) | 71.6 (67.1, 75.7) | 75.9 (72.1, 79.3) | 72.8 (67.3, 77.6) | 73.7 (71.4, 75.9) |
|  | Percentage Point Difference (95% CI) | -4.5 (-11.8, 2.8) | -1.2 (-6.9, 4.4) | 6.4* (1.5, 11.2) | 3.5 (-3.6, 10.7) | 1.7 (-1.3, 4.7) |
|  | Relative Difference (%) | -5.8 | -1.7 | 9.1* | 5.1 | 2.3 |
| $75,000-$99,999 | 2014-15 (%; 95% CI) | 72.7 (65.8, 78.7) | 68.9 (63.8, 73.6) | 68.1 (63, 72.9) | 69.2 (62.3, 75.3) | 69.5 (66.7, 72.2) |
|  | 2018-19 (%; 95% CI) | 75.2 (67.3, 81.8) | 69.8 (63.5, 75.4) | 73 (67.4, 78.1) | 67.9 (60.5, 74.4) | 71.4 (68.2, 74.5) |
|  | Percentage Point Difference (95% CI) | 2.5 (-7.2, 12.2) | 0.8 (-6.9, 8.6) | 4.9 (-2.4, 12.2) | -1.3 (-10.9, 8.2) | 1.9 (-2.3, 6.1) |
|  | Relative Difference (%) | 3.4 | 1.2 | 7.2 | -1.9 | 2.8 |
| $100,000-$149,999 | 2014-15 (%; 95% CI) | 75.1 (67.8, 81.2) | 72 (66.1, 77.3) | 67.4 (61.7, 72.6) | 63.6 (55.5, 71.1) | 69.4 (66.2, 72.5) |
|  | 2018-19 (%; 95% CI) | 69.1 (60.4, 76.7) | 66.8 (59.2, 73.6) | 65 (58.8, 70.7) | 73.4 (66, 79.8) | 68 (64.4, 71.4) |
|  | Percentage Point Difference (95% CI) | -6 (-16.6, 4.6) | -5.2 (-14.4, 4) | -2.4 (-10.5, 5.7) | 9.8 (-0.7, 20.3) | -1.4 (-6.1, 3.3) |
|  | Relative Difference (%) | -8 | -7.2 | -3.5 | 15.4 | -2 |
| $150,000+ | 2014-15 (%; 95% CI) | 75.2 (65.1, 83.2) | 75 (64.3, 83.4) | 70.3 (62.3, 77.1) | 74.7 (65.5, 82.1) | 73.5 (68.9, 77.5) |
|  | 2018-19 (%; 95% CI) | 81.1 (71.8, 87.9) | 70.5 (60.3, 79) | 62.4 (53.3, 70.7) | 69.6 (58.7, 78.7) | 69.5 (64.5, 74) |
|  | Percentage Point Difference (95% CI) | 5.9 (-6.2, 18) | -4.5 (-18, 8.9) | -7.9 (-19.3, 3.6) | -5 (-18.1, 8.1) | -4 (-10.4, 2.5) |
|  | Relative Difference (%) | 7.8 | -6 | -11.2 | -6.8 | -5.4 |

We indicate the level of significance for differences between survey years as follows: ^***^p<0.001, ^**^p<0.01, and ^*^p<0.05. The relative difference between survey years is calculated as: $\frac{Prevalence Estimate [2018-19] - Prevalence Estimate [2014-15]}{Prevalence Estimate [2014-15]}$.

# Appendix Table 6. Changes in Regional and National Quit Attempts by Demographic Group, US Adults (18+) – TUS-CPS 2014-15 and 2018-19

| Measure | Year | Region | | | | National |
| --- | --- | --- | --- | --- | --- | --- |
|  |  | Northeast | Midwest | South | West |  |
| **Overall** | **2014-15 (%; 95% CI)** | **54.2 (52.3, 56.1)** | **54.1 (52.7, 55.5)** | **52.3 (51.1, 53.5)** | **54.7 (52.9, 56.4)** | **53.5 (52.7, 54.2)** |
|  | **2018-19 (%; 95% CI)** | **55.8 (53.5, 58.1)** | **52 (50.2, 53.8)** | **49.5 (48.1, 50.9)** | **53.8 (51.7, 55.8)** | **51.9 (51, 52.8)** |
|  | **Percentage Point Difference (95% CI)** | **1.6 (-1.4, 4.6)** | **-2.1 (-4.4, 0.2)** | **-2.8 (-4.7, -1)**** | **-0.9 (-3.6, 1.8)** | **-1.6 (-2.7, -0.4)**** |
|  | **Relative Difference (%)** | **3** | **-3.8** | **-5.4**** | **-1.6** | **-2.9**** |
| Sex | | | | | | |
| Male | 2014-15 (%; 95% CI) | 51.2 (48.4, 54) | 53.8 (51.8, 55.9) | 50.4 (48.7, 52) | 53.6 (51.2, 55.9) | 52 (50.9, 53) |
|  | 2018-19 (%; 95% CI) | 53.9 (50.5, 57.3) | 49.7 (47.2, 52.2) | 46.2 (44.2, 48.2) | 52 (49.1, 54.8) | 49.4 (48.1, 50.6) |
|  | Percentage Point Difference (95% CI) | 2.7 (-1.7, 7.1) | -4.2* (-7.4, -0.9) | -4.2** (-6.8, -1.6) | -1.6 (-5.3, 2.1) | -2.6** (-4.2, -0.9) |
|  | Relative Difference (%) | 5.3 | -7.7* | -8.3** | -3 | -5** |
| Female | 2014-15 (%; 95% CI) | 57.6 (55, 60.3) | 54.3 (52.4, 56.3) | 54.5 (52.9, 56.1) | 56.1 (53.6, 58.5) | 55.3 (54.2, 56.3) |
|  | 2018-19 (%; 95% CI) | 57.8 (54.6, 61) | 54.6 (52.1, 57.1) | 53.5 (51.5, 55.4) | 56.3 (53.4, 59.2) | 55 (53.7, 56.2) |
|  | Percentage Point Difference (95% CI) | 0.2 (-4, 4.3) | 0.3 (-2.9, 3.4) | -1.1 (-3.6, 1.5) | 0.2 (-3.6, 4.1) | -0.3 (-1.9, 1.3) |
|  | Relative Difference (%) | 0.3 | 0.5 | -2 | 0.4 | -0.5 |
| Age Category, Years | | | | | | |
| 18–24 | 2014-15 (%; 95% CI) | 52.6 (44.7, 60.5) | 63.1 (58, 68) | 60.9 (56.7, 64.9) | 65.5 (59.8, 70.9) | 61.2 (58.6, 63.8) |
|  | 2018-19 (%; 95% CI) | 58.8 (45.9, 70.6) | 65.9 (57.4, 73.6) | 63.8 (57.5, 69.6) | 65.9 (57.3, 73.6) | 64.2 (60.1, 68.1) |
|  | Percentage Point Difference (95% CI) | 6.1 (-8.8, 21.1) | 2.8 (-6.8, 12.4) | 2.9 (-4.5, 10.2) | 0.4 (-9.6, 10.3) | 3 (-1.8, 7.8) |
|  | Relative Difference (%) | 11.6 | 4.4 | 4.7 | 0.6 | 4.9 |
| 25–34 | 2014-15 (%; 95% CI) | 59.9 (55.6, 64) | 60.3 (57.2, 63.3) | 56.4 (53.9, 59) | 56 (52.2, 59.7) | 57.9 (56.3, 59.5) |
|  | 2018-19 (%; 95% CI) | 61.3 (55.8, 66.5) | 58.3 (54.1, 62.4) | 55.5 (52.2, 58.8) | 60.4 (55.5, 65.1) | 58.1 (56, 60.2) |
|  | Percentage Point Difference (95% CI) | 1.4 (-5.4, 8.2) | -2 (-7.1, 3.1) | -0.9 (-5, 3.2) | 4.5 (-1.6, 10.6) | 0.2 (-2.4, 2.8) |
|  | Relative Difference (%) | 2.3 | -3.3 | -1.6 | 8 | 0.3 |
| 35–44 | 2014-15 (%; 95% CI) | 55 (50.6, 59.3) | 51.7 (48.5, 54.9) | 52.7 (50, 55.4) | 57.6 (53.8, 61.4) | 53.7 (52.1, 55.4) |
|  | 2018-19 (%; 95% CI) | 51.5 (46, 57) | 51.6 (47.6, 55.6) | 45.8 (42.7, 48.9) | 52.5 (48.1, 56.9) | 49.5 (47.5, 51.5) |
|  | Percentage Point Difference (95% CI) | -3.5 (-10.5, 3.5) | -0.1 (-5.3, 5) | -6.9** (-11, -2.8) | -5.1 (-10.9, 0.7) | -4.3** (-6.9, -1.7) |
|  | Relative Difference (%) | -6.3 | -0.3 | -13.1** | -8.9 | -8** |
| 45-64 | 2014-15 (%; 95% CI) | 50.7 (47.8, 53.5) | 50.1 (47.9, 52.3) | 48.3 (46.5, 50) | 50.8 (48.2, 53.4) | 49.6 (48.4, 50.7) |
|  | 2018-19 (%; 95% CI) | 55.5 (52.1, 58.9) | 46.8 (44.2, 49.4) | 46.1 (44, 48.2) | 50.5 (47.4, 53.7) | 48.7 (47.3, 50) |
|  | Percentage Point Difference (95% CI) | 4.9* (0.4, 9.3) | -3.3 (-6.7, 0.1) | -2.1 (-4.9, 0.6) | -0.3 (-4.4, 3.9) | -0.9 (-2.6, 0.8) |
|  | Relative Difference (%) | 9.6* | -6.5 | -4.4 | -0.5 | -1.8 |
| 65+ | 2014-15 (%; 95% CI) | 56.1 (50.9, 61.2) | 48.3 (44.4, 52.3) | 47.7 (44.6, 50.8) | 45.1 (40.5, 49.7) | 48.7 (46.7, 50.7) |
|  | 2018-19 (%; 95% CI) | 52.6 (47.1, 58) | 49.8 (45.5, 54) | 45.6 (42.4, 48.8) | 45 (40.4, 49.7) | 47.6 (45.5, 49.7) |
|  | Percentage Point Difference (95% CI) | -3.5 (-11, 4) | 1.4 (-4.4, 7.2) | -2.1 (-6.5, 2.4) | -0.1 (-6.6, 6.5) | -1.1 (-4, 1.8) |
|  | Relative Difference (%) | -6.3 | 3 | -4.4 | -0.2 | -2.3 |
| Race/Ethnicity | | | | | | |
| White, non-Hispanic | 2014-15 (%; 95% CI) | 53.5 (51.4, 55.7) | 52.9 (51.4, 54.5) | 50.8 (49.4, 52.1) | 54.8 (52.8, 56.8) | 52.5 (51.7, 53.3) |
|  | 2018-19 (%; 95% CI) | 54.5 (51.9, 57) | 50.9 (49, 52.8) | 47.9 (46.3, 49.5) | 51.9 (49.5, 54.3) | 50.5 (49.5, 51.5) |
|  | Percentage Point Difference (95% CI) | 0.9 (-2.4, 4.3) | -2 (-4.4, 0.5) | -2.9** (-5, -0.8) | -2.8 (-6, 0.3) | -2** (-3.3, -0.6) |
|  | Relative Difference (%) | 1.7 | -3.8 | -5.7** | -5.2 | -3.7** |
| Black, non-Hispanic | 2014-15 (%; 95% CI) | 56 (49.6, 62.2) | 59.5 (54.5, 64.3) | 54.4 (51.5, 57.3) | 54.5 (46.3, 62.5) | 55.7 (53.4, 57.9) |
|  | 2018-19 (%; 95% CI) | 67.3 (59.5, 74.3) | 55.9 (49.2, 62.3) | 53 (49.5, 56.6) | 61.3 (50.7, 70.9) | 56.3 (53.5, 59.1) |
|  | Percentage Point Difference (95% CI) | 11.3* (1.6, 21.1) | -3.7 (-11.9, 4.6) | -1.4 (-6, 3.2) | 6.8 (-6.3, 19.9) | 0.6 (-3, 4.2) |
|  | Relative Difference (%) | 20.2* | -6.1 | -2.5 | 12.4 | 1.1 |
| Hispanic | 2014-15 (%; 95% CI) | 59.1 (52.2, 65.7) | 55.5 (48, 62.7) | 54.4 (50.1, 58.6) | 55.5 (51.1, 59.8) | 55.7 (53.1, 58.3) |
|  | 2018-19 (%; 95% CI) | 53.3 (44.3, 62) | 56.9 (47.2, 66.1) | 49.9 (44.7, 55.1) | 54.7 (49.5, 59.7) | 52.9 (49.7, 56.1) |
|  | Percentage Point Difference (95% CI) | -5.8 (-17.1, 5.4) | 1.5 (-10.6, 13.5) | -4.5 (-11.2, 2.2) | -0.8 (-7.5, 5.9) | -2.8 (-6.9, 1.3) |
|  | Relative Difference (%) | -9.9 | 2.6 | -8.3 | -1.5 | -5.1 |
| Asian, non-Hispanic | 2014-15 (%; 95% CI) | 46.4 (34.8, 58.5) | 57.8 (44.6, 69.9) | 63.4 (52.8, 72.8) | 48.2 (40.4, 56.1) | 52.7 (47.5, 57.8) |
|  | 2018-19 (%; 95% CI) | 55.9 (42, 69) | 55.1 (38.8, 70.4) | 52.2 (40.7, 63.4) | 55.9 (46.8, 64.5) | 54.9 (49, 60.7) |
|  | Percentage Point Difference (95% CI) | 9.5 (-8.9, 27.8) | -2.7 (-23.5, 18.2) | -11.2 (-26.6, 4.1) | 7.6 (-4.3, 19.6) | 2.2 (-5.6, 10.1) |
|  | Relative Difference (%) | 20.4 | -4.6 | -17.7 | 15.9 | 4.2 |
| Other, non-Hispanic | 2014-15 (%; 95% CI) | 56.4 (40.3, 71.2) | 63.8 (55.4, 71.4) | 62.2 (54.7, 69.2) | 57.3 (50.4, 63.9) | 60.3 (56.1, 64.4) |
|  | 2018-19 (%; 95% CI) | 53 (36.8, 68.5) | 58.1 (46.3, 69.1) | 58.2 (50.2, 65.7) | 59.2 (51.3, 66.7) | 58.1 (53.2, 62.8) |
|  | Percentage Point Difference (95% CI) | -3.4 (-26.3, 19.5) | -5.7 (-19.8, 8.5) | -4 (-14.7, 6.6) | 1.9 (-8.3, 12.2) | -2.3 (-8.6, 4.1) |
|  | Relative Difference (%) | -6.1 | -8.9 | -6.5 | 3.4 | -3.8 |
| Education | | | | | | |
| 12th Grade or below (No Diploma) | 2014-15 (%; 95% CI) | 55 (49.8, 60.2) | 52.4 (48.4, 56.4) | 47.5 (44.8, 50.2) | 51.8 (47.1, 56.6) | 50.4 (48.5, 52.3) |
|  | 2018-19 (%; 95% CI) | 58 (51.4, 64.2) | 45.4 (40, 50.8) | 45.8 (42.4, 49.2) | 52.3 (46, 58.5) | 48.7 (46.2, 51.1) |
|  | Percentage Point Difference (95% CI) | 2.9 (-5.4, 11.2) | -7.1* (-13.8, -0.3) | -1.7 (-6.1, 2.6) | 0.4 (-7.5, 8.3) | -1.7 (-4.8, 1.4) |
|  | Relative Difference (%) | 5.3 | -13.5* | -3.6 | 0.8 | -3.4 |
| Graduation from high school | 2014-15 (%; 95% CI) | 51.6 (48.3, 54.8) | 49.5 (47, 52) | 50.9 (48.7, 53) | 50.3 (47, 53.6) | 50.6 (49.2, 51.9) |
|  | 2018-19 (%; 95% CI) | 52.5 (48.5, 56.5) | 50.3 (47.2, 53.4) | 46.4 (43.9, 48.9) | 49 (45, 53.1) | 48.8 (47.2, 50.5) |
|  | Percentage Point Difference (95% CI) | 0.9 (-4.2, 6.1) | 0.8 (-3.2, 4.8) | -4.5** (-7.8, -1.2) | -1.3 (-6.5, 4) | -1.7 (-3.8, 0.4) |
|  | Relative Difference (%) | 1.8 | 1.5 | -8.8** | -2.6 | -3.4 |
| GED or other equivalent | 2014-15 (%; 95% CI) | 57.4 (49.7, 64.8) | 57.9 (52.1, 63.4) | 51 (46.9, 55.1) | 55.3 (48.9, 61.4) | 54.3 (51.5, 57) |
|  | 2018-19 (%; 95% CI) | 55.3 (45.8, 64.4) | 56.7 (49.8, 63.5) | 49.6 (44.5, 54.7) | 51.6 (43.5, 59.7) | 52.6 (49.1, 56) |
|  | Percentage Point Difference (95% CI) | -2.1 (-14.2, 10) | -1.1 (-10, 7.8) | -1.4 (-7.9, 5.2) | -3.7 (-13.9, 6.6) | -1.7 (-6.1, 2.7) |
|  | Relative Difference (%) | -3.7 | -1.9 | -2.7 | -6.6 | -3.1 |
| Some College or Above | 2014-15 (%; 95% CI) | 55.5 (52.6, 58.4) | 57.2 (55.1, 59.2) | 55.6 (53.9, 57.4) | 57.6 (55.2, 59.9) | 56.4 (55.3, 57.5) |
|  | 2018-19 (%; 95% CI) | 57.6 (54.2, 61) | 54.3 (51.8, 56.7) | 53.1 (51.1, 55.2) | 56.8 (54.1, 59.5) | 54.9 (53.6, 56.2) |
|  | Percentage Point Difference (95% CI) | 2.1 (-2.3, 6.6) | -2.9 (-6.2, 0.3) | -2.5 (-5.2, 0.2) | -0.8 (-4.4, 2.8) | -1.5 (-3.2, 0.2) |
|  | Relative Difference (%) | 3.8 | -5.1 | -4.5 | -1.4 | -2.7 |
| Marital Status | | | | | | |
| Not Married | 2014-15 (%; 95% CI) | 54.7 (52.2, 57.2) | 53.8 (52, 55.7) | 51.4 (49.9, 52.9) | 54.5 (52.3, 56.7) | 53.2 (52.2, 54.1) |
|  | 2018-19 (%; 95% CI) | 56.7 (53.8, 59.5) | 52.8 (50.5, 55.2) | 50.7 (48.9, 52.5) | 55.2 (52.6, 57.8) | 53.1 (51.9, 54.2) |
|  | Percentage Point Difference (95% CI) | 2 (-1.8, 5.7) | -1 (-4, 2) | -0.7 (-3.1, 1.6) | 0.7 (-2.7, 4.1) | -0.1 (-1.6, 1.4) |
|  | Relative Difference (%) | 3.6 | -1.8 | -1.4 | 1.3 | -0.1 |
| Married | 2014-15 (%; 95% CI) | 53.2 (50.2, 56.3) | 54.5 (52.2, 56.7) | 53.8 (52, 55.6) | 55 (52.2, 57.7) | 54.1 (52.9, 55.2) |
|  | 2018-19 (%; 95% CI) | 54.2 (50.2, 58.1) | 50.5 (47.8, 53.3) | 47.5 (45.3, 49.8) | 51.3 (47.9, 54.6) | 50 (48.5, 51.4) |
|  | Percentage Point Difference (95% CI) | 0.9 (-4.1, 5.9) | -3.9* (-7.5, -0.4) | -6.2*** (-9.1, -3.4) | -3.7 (-8, 0.6) | -4.1*** (-6, -2.3) |
|  | Relative Difference (%) | 1.7 | -7.2* | -11.6*** | -6.7 | -7.6*** |
| Employment Status | | | | | | |
| Not Working | 2014-15 (%; 95% CI) | 58.8 (55.8, 61.7) | 53.5 (51.1, 55.7) | 50.9 (49.1, 52.6) | 54 (51.3, 56.6) | 53.3 (52.2, 54.4) |
|  | 2018-19 (%; 95% CI) | 55.4 (51.8, 59) | 52.9 (50, 55.8) | 49.4 (47.3, 51.4) | 51.2 (48, 54.4) | 51.5 (50.1, 52.9) |
|  | Percentage Point Difference (95% CI) | -3.4 (-8.1, 1.3) | -0.6 (-4.3, 3.1) | -1.5 (-4.2, 1.2) | -2.7 (-6.9, 1.4) | -1.8* (-3.6, 0) |
|  | Relative Difference (%) | -5.8 | -1 | -2.9 | -5.1 | -3.4* |
| Working | 2014-15 (%; 95% CI) | 51.1 (48.6, 53.7) | 54.4 (52.6, 56.3) | 53.4 (51.8, 55) | 55.1 (52.8, 57.4) | 53.6 (52.6, 54.6) |
|  | 2018-19 (%; 95% CI) | 56.1 (53, 59.1) | 51.5 (49.2, 53.7) | 49.5 (47.6, 51.4) | 55.3 (52.7, 58) | 52.2 (51, 53.4) |
|  | Percentage Point Difference (95% CI) | 5* (1, 8.9) | -3* (-5.9, -0.1) | -3.9** (-6.4, -1.4) | 0.2 (-3.2, 3.7) | -1.4 (-2.9, 0.1) |
|  | Relative Difference (%) | 9.7* | -5.4* | -7.3** | 0.4 | -2.6 |
| Annual Household Income, $USD | | | | | | |
| Below $25,000 | 2014-15 (%; 95% CI) | 55.7 (52.2, 59.2) | 54.6 (52.1, 57.1) | 50.7 (48.9, 52.6) | 55.4 (52.4, 58.3) | 53.2 (51.9, 54.4) |
|  | 2018-19 (%; 95% CI) | 57.2 (52.7, 61.6) | 54.7 (51.3, 58.1) | 51 (48.6, 53.3) | 51.8 (48, 55.7) | 53 (51.3, 54.6) |
|  | Percentage Point Difference (95% CI) | 1.5 (-4.2, 7.1) | 0.1 (-4.2, 4.4) | 0.2 (-2.8, 3.2) | -3.6 (-8.4, 1.3) | -0.2 (-2.3, 1.8) |
|  | Relative Difference (%) | 2.7 | 0.2 | 0.4 | -6.4 | -0.4 |
| $25,000-$49,999 | 2014-15 (%; 95% CI) | 52.8 (49.1, 56.6) | 53.8 (51.1, 56.4) | 53.2 (51, 55.3) | 52.9 (49.8, 56.1) | 53.2 (51.8, 54.6) |
|  | 2018-19 (%; 95% CI) | 53.5 (48.8, 58) | 49.4 (46.2, 52.7) | 49.2 (46.6, 51.8) | 55.3 (51.3, 59.1) | 50.9 (49.3, 52.6) |
|  | Percentage Point Difference (95% CI) | 0.6 (-5.3, 6.6) | -4.3* (-8.5, -0.2) | -4* (-7.4, -0.5) | 2.3 (-2.7, 7.4) | -2.3* (-4.5, -0.1) |
|  | Relative Difference (%) | 1.2 | -8* | -7.5* | 4.4 | -4.3* |
| $50,000-$74,999 | 2014-15 (%; 95% CI) | 50.6 (46.2, 55.1) | 53.5 (50.2, 56.8) | 52.6 (49.7, 55.6) | 54.8 (50.6, 58.9) | 52.9 (51.1, 54.7) |
|  | 2018-19 (%; 95% CI) | 54.8 (49.4, 60.1) | 51.2 (47.3, 55.1) | 48.2 (44.9, 51.6) | 54.1 (49.4, 58.8) | 51.2 (49.1, 53.2) |
|  | Percentage Point Difference (95% CI) | 4.1 (-2.8, 11.1) | -2.3 (-7.4, 2.8) | -4.4 (-8.8, 0) | -0.6 (-6.9, 5.6) | -1.7 (-4.5, 1) |
|  | Relative Difference (%) | 8.2 | -4.3 | -8.4 | -1.2 | -3.3 |
| $75,000-$99,999 | 2014-15 (%; 95% CI) | 52.8 (46.9, 58.6) | 54.5 (49.9, 59.1) | 51.4 (47.1, 55.7) | 54 (48.2, 59.7) | 53 (50.5, 55.5) |
|  | 2018-19 (%; 95% CI) | 60.5 (53.5, 67.2) | 55.4 (50, 60.7) | 48.6 (43.7, 53.6) | 53.7 (47.6, 59.8) | 53.7 (50.8, 56.5) |
|  | Percentage Point Difference (95% CI) | 7.7 (-1.3, 16.8) | 0.9 (-6.2, 8) | -2.8 (-9.3, 3.8) | -0.3 (-8.7, 8.2) | 0.6 (-3.2, 4.4) |
|  | Relative Difference (%) | 14.6 | 1.7 | -5.4 | -0.5 | 1.2 |
| $100,000-$149,999 | 2014-15 (%; 95% CI) | 56.9 (49.9, 63.7) | 54.7 (49.3, 60) | 60.3 (55.4, 65) | 59.5 (52.9, 65.7) | 58 (55.1, 60.9) |
|  | 2018-19 (%; 95% CI) | 53.8 (46.7, 60.7) | 52.4 (46.1, 58.7) | 50.4 (45.3, 55.6) | 57.3 (50.6, 63.7) | 53.1 (50, 56.2) |
|  | Percentage Point Difference (95% CI) | -3.1 (-13, 6.8) | -2.3 (-10.6, 6) | -9.9** (-16.9, -2.8) | -2.2 (-11.4, 7.1) | -4.9* (-9.1, -0.7) |
|  | Relative Difference (%) | -5.5 | -4.1 | -16.4** | -3.6 | -8.4* |
| $150,000+ | 2014-15 (%; 95% CI) | 62.5 (54.4, 69.9) | 51.8 (42.8, 60.7) | 50.1 (43.1, 57.2) | 52.2 (43.8, 60.4) | 54.3 (50.2, 58.3) |
|  | 2018-19 (%; 95% CI) | 57.8 (48.6, 66.6) | 45.6 (37.1, 54.3) | 46.3 (39.2, 53.5) | 49 (40.3, 57.7) | 49.1 (45, 53.3) |
|  | Percentage Point Difference (95% CI) | -4.6 (-16.6, 7.4) | -6.2 (-18.8, 6.4) | -3.9 (-13.9, 6.2) | -3.2 (-15.3, 9) | -5.2 (-11, 0.7) |
|  | Relative Difference (%) | -7.4 | -12 | -7.7 | -6.1 | -9.5 |

We indicate the level of significance for differences between survey years as follows: ^***^p<0.001, ^**^p<0.01, and ^*^p<0.05. The relative difference between survey years is calculated as: $\frac{Prevalence Estimate [2018-19] - Prevalence Estimate [2014-15]}{Prevalence Estimate [2014-15]}$.

# Appendix Table 7. Changes in Regional and National Recent Successful Quitting by Demographic Group, US Adults (18+) – TUS-CPS 2014-15 and 2018-19

| Measure | Year | Region | | | | National |
| --- | --- | --- | --- | --- | --- | --- |
|  |  | Northeast | Midwest | South | West |  |
| **Overall** | **2014-15 (%; 95% CI)** | **7.1 (6.2, 8.1)** | **7.8 (7, 8.6)** | **7 (6.4, 7.7)** | **8.9 (7.9, 10)** | **7.6 (7.2, 8)** |
|  | **2018-19 (%; 95% CI)** | **7.5 (6.3, 8.9)** | **7.5 (6.6, 8.5)** | **6.9 (6.2, 7.7)** | **8.2 (7.2, 9.5)** | **7.4 (6.9, 7.9)** |
|  | **Percentage Point Difference (95% CI)** | **0.4 (-1.2, 2)** | **-0.3 (-1.6, 0.9)** | **-0.1 (-1, 0.9)** | **-0.7 (-2.2, 0.9)** | **-0.2 (-0.8, 0.5)** |
|  | **Relative Difference (%)** | **5.6** | **-4** | **-1.3** | **-7.5** | **-2.1** |
| Sex | | | | | | |
| Male | 2014-15 (%; 95% CI) | 6.6 (5.4, 8.1) | 8.4 (7.3, 9.7) | 6.6 (5.8, 7.5) | 8.6 (7.3, 10.1) | 7.4 (6.9, 8) |
|  | 2018-19 (%; 95% CI) | 7.9 (6.2, 10) | 7.5 (6.3, 8.9) | 6.6 (5.6, 7.6) | 8.2 (6.8, 9.9) | 7.3 (6.7, 8) |
|  | Percentage Point Difference (95% CI) | 1.3 (-1, 3.6) | -0.9 (-2.7, 0.9) | 0 (-1.4, 1.3) | -0.4 (-2.4, 1.7) | -0.1 (-1, 0.8) |
|  | Relative Difference (%) | 19.7 | -10.7 | -0.8 | -4.2 | -1.2 |
| Female | 2014-15 (%; 95% CI) | 7.6 (6.3, 9.2) | 7.1 (6.2, 8.2) | 7.5 (6.7, 8.5) | 9.3 (7.9, 11) | 7.7 (7.2, 8.3) |
|  | 2018-19 (%; 95% CI) | 7 (5.4, 9.1) | 7.5 (6.2, 9) | 7.4 (6.4, 8.5) | 8.2 (6.6, 10.2) | 7.5 (6.8, 8.2) |
|  | Percentage Point Difference (95% CI) | -0.6 (-2.9, 1.7) | 0.3 (-1.4, 2.1) | -0.1 (-1.5, 1.3) | -1.1 (-3.4, 1.3) | -0.2 (-1.1, 0.7) |
|  | Relative Difference (%) | -7.7 | 4.8 | -1.6 | -11.5 | -3 |
| Age Category, Years | | | | | | |
| 18–24 | 2014-15 (%; 95% CI) | 7.3 (4.4, 12.1) | 12.4 (9.2, 16.6) | 10.1 (7.7, 13.2) | 13.1 (9.4, 18) | 10.9 (9.3, 12.8) |
|  | 2018-19 (%; 95% CI) | 12.8 (6.1, 25.2)*RSE | 14.2 (9.1, 21.4) | 14.7 (10.9, 19.5) | 14.7 (9.8, 21.5) | 14.4 (11.7, 17.5) |
|  | Percentage Point Difference (95% CI) | 5.5 (-4.5, 15.5) | 1.8 (-5.3, 8.9) | 4.6 (-0.5, 9.7) | 1.7 (-5.6, 8.9) | 3.5* (0.1, 6.8) |
|  | Relative Difference (%) | 74.8 | 14.4 | 45.4 | 12.7 | 31.8* |
| 25–34 | 2014-15 (%; 95% CI) | 10 (7.6, 12.9) | 10.4 (8.7, 12.5) | 8.5 (7.2, 10.1) | 11.9 (9.6, 14.7) | 9.9 (8.9, 10.9) |
|  | 2018-19 (%; 95% CI) | 9.9 (7.1, 13.8) | 10.8 (8.5, 13.5) | 8.7 (7, 10.8) | 11.1 (8.5, 14.4) | 9.9 (8.7, 11.2) |
|  | Percentage Point Difference (95% CI) | 0 (-4.3, 4.2) | 0.3 (-2.8, 3.5) | 0.2 (-2.2, 2.6) | -0.8 (-4.7, 3) | 0 (-1.6, 1.6) |
|  | Relative Difference (%) | -0.3 | 3.1 | 2.5 | -6.9 | 0.4 |
| 35–44 | 2014-15 (%; 95% CI) | 6.7 (4.8, 9.1) | 6.6 (5.2, 8.3) | 7.3 (6, 8.8) | 8.3 (6.4, 10.7) | 7.2 (6.4, 8.1) |
|  | 2018-19 (%; 95% CI) | 9.6 (6.6, 13.6) | 6 (4.3, 8.3) | 7.2 (5.8, 9) | 9.1 (6.7, 12.3) | 7.6 (6.6, 8.8) |
|  | Percentage Point Difference (95% CI) | 2.9 (-1.1, 7) | -0.6 (-3.1, 1.9) | 0 (-2.1, 2.1) | 0.8 (-2.7, 4.3) | 0.4 (-1, 1.8) |
|  | Relative Difference (%) | 43.9 | -9.5 | -0.4 | 9.6 | 6.1 |
| 45-64 | 2014-15 (%; 95% CI) | 5.7 (4.5, 7.1) | 5.6 (4.7, 6.7) | 5.6 (4.8, 6.5) | 6.4 (5.2, 7.8) | 5.7 (5.2, 6.3) |
|  | 2018-19 (%; 95% CI) | 5.1 (3.8, 6.8) | 5.5 (4.5, 6.8) | 4.6 (3.8, 5.6) | 5.5 (4.2, 7.2) | 5.1 (4.5, 5.7) |
|  | Percentage Point Difference (95% CI) | -0.6 (-2.6, 1.4) | -0.1 (-1.6, 1.4) | -1 (-2.2, 0.2) | -0.9 (-2.8, 1.1) | -0.6 (-1.4, 0.1) |
|  | Relative Difference (%) | -10.6 | -1.4 | -17.2 | -13.5 | -11.2 |
| 65+ | 2014-15 (%; 95% CI) | 7.1 (4.9, 10.1) | 6.9 (5.2, 9.2) | 5.6 (4.4, 7.2) | 7.5 (5.4, 10.4) | 6.5 (5.6, 7.6) |
|  | 2018-19 (%; 95% CI) | 6.2 (3.9, 9.6) | 6.8 (5, 9.1) | 5.7 (4.4, 7.4) | 5.8 (4, 8.3) | 6 (5.1, 7.1) |
|  | Percentage Point Difference (95% CI) | -0.9 (-4.6, 2.9) | -0.2 (-3, 2.7) | 0.1 (-2, 2.1) | -1.7 (-5, 1.5) | -0.5 (-1.9, 0.9) |
|  | Relative Difference (%) | -12.7 | -2.7 | 0.9 | -23.2 | -7.4 |
| Race/Ethnicity | | | | | | |
| White, non-Hispanic | 2014-15 (%; 95% CI) | 7.7 (6.6, 8.9) | 7.9 (7.1, 8.8) | 7.6 (6.9, 8.3) | 9.2 (8, 10.5) | 7.9 (7.5, 8.4) |
|  | 2018-19 (%; 95% CI) | 7.3 (6, 8.8) | 7.3 (6.4, 8.4) | 7 (6.3, 7.9) | 8 (6.8, 9.5) | 7.3 (6.8, 7.9) |
|  | Percentage Point Difference (95% CI) | -0.4 (-2.2, 1.4) | -0.6 (-1.9, 0.7) | -0.5 (-1.6, 0.6) | -1.1 (-3, 0.7) | -0.6 (-1.3, 0.1) |
|  | Relative Difference (%) | -5.4 | -7.4 | -6.9 | -12.4 | -7.7 |
| Black, non-Hispanic | 2014-15 (%; 95% CI) | 3.8 (2, 7.2)*RSE | 4.9 (3, 7.9) | 4.6 (3.5, 6.1) | 8.2 (4.8, 13.5) | 4.8 (3.9, 6) |
|  | 2018-19 (%; 95% CI) | 6 (3.1, 11.2)*RSE | 3.7 (1.9, 7)*RSE | 5.3 (3.8, 7.4) | 7 (3.3, 14)*RSE | 5.2 (4, 6.7) |
|  | Percentage Point Difference (95% CI) | 2.2 (-2.4, 6.8) | -1.2 (-4.5, 2.2) | 0.7 (-1.5, 2.9) | -1.2 (-7.8, 5.4) | 0.4 (-1.3, 2) |
|  | Relative Difference (%) | 58.9 | -24.2 | 14.6 | -14.7 | 7.4 |
| Hispanic | 2014-15 (%; 95% CI) | 7.8 (4.7, 12.7) | 12.9 (8.4, 19.4) | 7.7 (5.6, 10.5) | 8.6 (6.4, 11.5) | 8.6 (7.2, 10.3) |
|  | 2018-19 (%; 95% CI) | 6.5 (3.3, 12.5)*RSE | 17.5 (11.1, 26.5) | 8 (5.6, 11.3) | 9.6 (6.9, 13.2) | 9.4 (7.7, 11.5) |
|  | Percentage Point Difference (95% CI) | -1.2 (-7.1, 4.6) | 4.5 (-4.9, 13.9) | 0.3 (-3.4, 4) | 0.9 (-3.1, 5) | 0.8 (-1.7, 3.3) |
|  | Relative Difference (%) | -16 | 35.1 | 4 | 10.9 | 9.3 |
| Asian, non-Hispanic | 2014-15 (%; 95% CI) | 3.7 (1.1, 11.4)*RSE | 8.3 (3.7, 17.4)*RSE | 7.8 (3.3, 17.2)*RSE | 8 (4.5, 13.8) | 7 (4.8, 10.3) |
|  | 2018-19 (%; 95% CI) | 17.4 (7.9, 34.1)*RSE | 10.2 (4, 23.7)*RSE | 9.7 (4.5, 19.5)*RSE | 7.6 (4, 14.1)*RSE | 10.2 (7, 14.8) |
|  | Percentage Point Difference (95% CI) | 13.7* (0.1, 27.3) | 2 (-9.3, 13.2) | 1.9 (-7.7, 11.5) | -0.3 (-6.9, 6.3) | 3.2 (-1.5, 7.9) |
|  | Relative Difference (%) | 369.2* | 23.6 | 24.4 | -4.1 | 45 |
| Other, non-Hispanic | 2014-15 (%; 95% CI) | 5.4 (1.4, 18.4)*RSE | 6.5 (3.6, 11.3) | 5.4 (2.5, 11.2)*RSE | 9 (5.4, 14.6) | 6.8 (4.8, 9.5) |
|  | 2018-19 (%; 95% CI) | 11.6 (4.3, 27.7)*RSE | 9.6 (4.6, 19)*RSE | 8.6 (5.1, 14.1) | 7.5 (4.6, 12) | 8.7 (6.4, 11.8) |
|  | Percentage Point Difference (95% CI) | 6.2 (-6.8, 19.2) | 3.2 (-4.7, 11) | 3.1 (-2.9, 9.1) | -1.5 (-7.2, 4.3) | 1.9 (-1.7, 5.4) |
|  | Relative Difference (%) | 115.3 | 48.5 | 57.5 | -16.4 | 27.7 |
| Education | | | | | | |
| 12th Grade or below (No Diploma) | 2014-15 (%; 95% CI) | 4.8 (3, 7.5) | 4.1 (2.8, 6.1) | 4 (3.1, 5.2) | 6.4 (4.5, 9) | 4.5 (3.8, 5.4) |
|  | 2018-19 (%; 95% CI) | 7.1 (4.3, 11.6) | 5.3 (3.3, 8.4) | 4.1 (2.9, 5.8) | 5.4 (3.3, 8.8) | 5.1 (4.1, 6.3) |
|  | Percentage Point Difference (95% CI) | 2.4 (-1.8, 6.5) | 1.2 (-1.8, 4.1) | 0.1 (-1.6, 1.9) | -1 (-4.5, 2.5) | 0.6 (-0.8, 1.9) |
|  | Relative Difference (%) | 49.5 | 28.2 | 3.1 | -15.4 | 12.3 |
| Graduation from high school | 2014-15 (%; 95% CI) | 6.3 (4.9, 8) | 6.3 (5.1, 7.6) | 6.6 (5.5, 7.8) | 7.7 (6.1, 9.8) | 6.6 (6, 7.4) |
|  | 2018-19 (%; 95% CI) | 6 (4.2, 8.3) | 6.3 (4.9, 8) | 5.8 (4.7, 7.2) | 7.9 (5.9, 10.5) | 6.3 (5.5, 7.2) |
|  | Percentage Point Difference (95% CI) | -0.3 (-2.9, 2.2) | 0 (-1.9, 2) | -0.8 (-2.5, 0.9) | 0.2 (-2.8, 3.1) | -0.3 (-1.4, 0.8) |
|  | Relative Difference (%) | -4.8 | 0.5 | -12.1 | 2.2 | -4.9 |
| GED or other equivalent | 2014-15 (%; 95% CI) | 6.1 (3.4, 10.7) | 5.6 (3.4, 9.1) | 3.1 (2, 4.8) | 6.2 (3.7, 10.3) | 4.7 (3.6, 6) |
|  | 2018-19 (%; 95% CI) | 4.3 (1.8, 9.8)*RSE | 4.5 (2.4, 8.5)*RSE | 3.5 (2, 5.9) | 3.8 (1.6, 8.3)*RSE | 3.9 (2.8, 5.5) |
|  | Percentage Point Difference (95% CI) | -1.8 (-6.8, 3.3) | -1.1 (-5.1, 2.9) | 0.3 (-2, 2.6) | -2.5 (-6.9, 2) | -0.8 (-2.5, 1) |
|  | Relative Difference (%) | -29.1 | -19.4 | 10.6 | -39.8 | -16.6 |
| Some College or Above | 2014-15 (%; 95% CI) | 8.7 (7.2, 10.4) | 10.3 (9, 11.7) | 9.4 (8.4, 10.5) | 10.5 (9.1, 12.2) | 9.7 (9.1, 10.4) |
|  | 2018-19 (%; 95% CI) | 9.1 (7.2, 11.4) | 9.2 (7.8, 10.8) | 9.4 (8.2, 10.7) | 9.7 (8.2, 11.5) | 9.4 (8.6, 10.2) |
|  | Percentage Point Difference (95% CI) | 0.4 (-2.2, 3.1) | -1 (-3, 1) | 0 (-1.6, 1.6) | -0.8 (-3.1, 1.4) | -0.4 (-1.4, 0.7) |
|  | Relative Difference (%) | 4.8 | -10.1 | 0 | -7.8 | -3.7 |
| Marital Status | | | | | | |
| Not Married | 2014-15 (%; 95% CI) | 6.7 (5.6, 8) | 6.9 (5.9, 7.9) | 6.3 (5.6, 7.2) | 8.5 (7.3, 9.8) | 6.9 (6.4, 7.4) |
|  | 2018-19 (%; 95% CI) | 7.3 (5.8, 9.1) | 6.9 (5.8, 8.2) | 6.7 (5.8, 7.7) | 8.2 (6.8, 9.8) | 7.1 (6.6, 7.8) |
|  | Percentage Point Difference (95% CI) | 0.6 (-1.4, 2.6) | 0.1 (-1.5, 1.6) | 0.4 (-0.9, 1.6) | -0.3 (-2.3, 1.6) | 0.2 (-0.6, 1) |
|  | Relative Difference (%) | 9.2 | 0.9 | 5.9 | -3.7 | 3.2 |
| Married | 2014-15 (%; 95% CI) | 7.9 (6.3, 9.8) | 9.3 (8.1, 10.7) | 8.1 (7.2, 9.2) | 9.7 (8.1, 11.6) | 8.7 (8, 9.4) |
|  | 2018-19 (%; 95% CI) | 7.9 (5.9, 10.4) | 8.4 (7, 10.1) | 7.3 (6.2, 8.5) | 8.4 (6.7, 10.4) | 7.9 (7.1, 8.7) |
|  | Percentage Point Difference (95% CI) | 0 (-2.8, 2.8) | -0.9 (-2.9, 1.2) | -0.8 (-2.4, 0.7) | -1.3 (-3.8, 1.2) | -0.8 (-1.8, 0.2) |
|  | Relative Difference (%) | -0.1 | -9.5 | -10.2 | -13.6 | -9.1 |
| Employment Status | | | | | | |
| Not Working | 2014-15 (%; 95% CI) | 6.2 (4.9, 7.7) | 6.7 (5.6, 7.9) | 5.8 (5, 6.7) | 7.8 (6.4, 9.5) | 6.4 (5.9, 7) |
|  | 2018-19 (%; 95% CI) | 5.9 (4.4, 8) | 6.1 (4.9, 7.6) | 5.5 (4.7, 6.5) | 7.1 (5.5, 9) | 6 (5.4, 6.7) |
|  | Percentage Point Difference (95% CI) | -0.3 (-2.5, 2) | -0.6 (-2.4, 1.2) | -0.2 (-1.5, 1) | -0.8 (-3, 1.5) | -0.4 (-1.3, 0.5) |
|  | Relative Difference (%) | -4.1 | -10.9 | 2.9 | -24 | -7.3 |
| Working | 2014-15 (%; 95% CI) | 7.7 (6.5, 9.1) | 8.5 (7.5, 9.6) | 8 (7.2, 8.9) | 9.6 (8.3, 11.1) | 8.4 (7.8, 8.9) |
|  | 2018-19 (%; 95% CI) | 8.5 (6.9, 10.5) | 8.3 (7.1, 9.7) | 7.9 (6.9, 9) | 9 (7.5, 10.7) | 8.3 (7.7, 9) |
|  | Percentage Point Difference (95% CI) | 0.8 (-1.4, 3) | -0.1 (-1.8, 1.5) | -0.1 (-1.4, 1.3) | -0.7 (-2.7, 1.4) | 0 (-0.9, 0.8) |
|  | Relative Difference (%) | -22.8 | -29.7 | -25.7 | -38.3 | -29 |
| Annual Household Income, $USD | | | | | | |
| Below $25,000 | 2014-15 (%; 95% CI) | 3.9 (2.9, 5.3) | 5.4 (4.3, 6.7) | 5.2 (4.4, 6.2) | 8.1 (6.5, 10.1) | 5.6 (5, 6.2) |
|  | 2018-19 (%; 95% CI) | 7.3 (5.1, 10.3) | 6.1 (4.6, 7.9) | 4.9 (3.9, 6) | 6.4 (4.7, 8.6) | 5.8 (5, 6.6) |
|  | Percentage Point Difference (95% CI) | 3.3* (0.5, 6.2) | 0.7 (-1.3, 2.7) | -0.3 (-1.7, 1) | -1.8 (-4.4, 0.8) | 0.2 (-0.8, 1.2) |
|  | Relative Difference (%) | 84.9* | 34.6 | 39.1 | -11 | 30.2 |
| $25,000-$49,999 | 2014-15 (%; 95% CI) | 8.6 (6.7, 11) | 8.7 (7.3, 10.5) | 7 (5.9, 8.2) | 8 (6.4, 9.9) | 7.9 (7.1, 8.7) |
|  | 2018-19 (%; 95% CI) | 4 (2.6, 6.1) | 7.3 (5.6, 9.3) | 7.5 (6.1, 9) | 8.9 (6.8, 11.4) | 7.2 (6.3, 8.1) |
|  | Percentage Point Difference (95% CI) | -4.6*** (-7.3, -1.9) | -1.5 (-3.9, 1) | 0.5 (-1.4, 2.3) | 0.9 (-2, 3.7) | -0.7 (-1.9, 0.5) |
|  | Relative Difference (%) | -15.8*** | -17.1 | 3.9 | -9 | -7.9 |
| $50,000-$74,999 | 2014-15 (%; 95% CI) | 6.2 (4.5, 8.7) | 7.7 (6.1, 9.7) | 9 (7.4, 11) | 10.5 (8, 13.7) | 8.4 (7.5, 9.5) |
|  | 2018-19 (%; 95% CI) | 10.2 (7.2, 14.3) | 7.1 (5.3, 9.3) | 6.8 (5.4, 8.7) | 9.1 (6.5, 12.5) | 7.9 (6.8, 9.1) |
|  | Percentage Point Difference (95% CI) | 4 (-0.1, 8.1) | -0.7 (-3.4, 2) | -2.2 (-4.6, 0.2) | -1.4 (-5.5, 2.7) | -0.6 (-2.1, 1) |
|  | Relative Difference (%) | 16.1 | -6.4 | -19.8 | -30.9 | -14.1 |
| $75,000-$99,999 | 2014-15 (%; 95% CI) | 8 (5.4, 11.6) | 10.6 (8, 13.9) | 7.8 (5.9, 10.4) | 9.6 (6.7, 13.7) | 8.9 (7.6, 10.4) |
|  | 2018-19 (%; 95% CI) | 6.1 (3.2, 11.3)*RSE | 10.4 (7.5, 14.2) | 11 (8.1, 14.7) | 9.1 (6.1, 13.2) | 9.6 (8, 11.5) |
|  | Percentage Point Difference (95% CI) | -1.9 (-6.8, 3.1) | -0.2 (-4.6, 4.2) | 3.1 (-0.8, 7.1) | -0.6 (-5.5, 4.3) | 0.7 (-1.6, 2.9) |
|  | Relative Difference (%) | -8.8 | -31.5 | -7.3 | -24.8 | -18.7 |
| $100,000-$149,999 | 2014-15 (%; 95% CI) | 12.2 (8.2, 17.9) | 9.4 (6.7, 12.9) | 12.8 (9.8, 16.6) | 10.7 (7.5, 15.2) | 11.4 (9.6, 13.4) |
|  | 2018-19 (%; 95% CI) | 9.2 (6, 14) | 11 (7.5, 15.9) | 8.5 (6.1, 11.7) | 8.9 (5.8, 13.5) | 9.3 (7.7, 11.3) |
|  | Percentage Point Difference (95% CI) | -3 (-9.2, 3.2) | 1.7 (-3.5, 6.8) | -4.3 (-8.7, 0.1) | -1.8 (-7.2, 3.6) | -2 (-4.6, 0.6) |
|  | Relative Difference (%) | -40.6 | -22.7 | -43.3 | -32.4 | -36.2 |
| $150,000+ | 2014-15 (%; 95% CI) | 11.4 (7.2, 17.5) | 16.3 (10.5, 24.4) | 9.8 (6.5, 14.3) | 10.5 (6.3, 16.8) | 11.4 (9.1, 14.2) |
|  | 2018-19 (%; 95% CI) | 12.3 (7.3, 20) | 6.2 (3.3, 11.2)*RSE | 8.4 (5.4, 12.9) | 8.4 (4.9, 14) | 8.7 (6.7, 11.2) |
|  | Percentage Point Difference (95% CI) | 0.9 (-7.1, 9) | -10.1* (-18, -2.3) | -1.4 (-6.7, 4) | -2.1 (-8.8, 4.7) | -2.7 (-6.1, 0.6) |
|  | Relative Difference (%) | -36.2 | -55.5* | -25.6 | -30.7 | -36.5 |

We indicate the level of significance for differences between survey years as follows: ^***^p<0.001, ^**^p<0.01, and ^*^p<0.05. The relative difference between survey years is calculated as: $\frac{Prevalence Estimate [2018-19] - Prevalence Estimate [2014-15]}{Prevalence Estimate [2014-15]}$. Due to low precision, estimates with a relative standard error greater than 30% have been indicated by “*RSE” and should be interpreted with caution.
